# Human induced pluripotent stem cell-derived closed-loop cardiac tissue for drug assessment

**DOI:** 10.1016/j.isci.2024.108992

**Published:** 2024-01-23

**Authors:** Junjun Li, Ying Hua, Yuting Liu, Xiang Qu, Jingbo Zhang, Masako Ishida, Noriko Yoshida, Akiko Tabata, Hayato Miyoshi, Mikio Shiba, Shuichiro Higo, Nagako Sougawa, Maki Takeda, Takuji Kawamura, Ryohei Matsuura, Daisuke Okuzaki, Toshihiko Toyofuku, Yoshiki Sawa, Li Liu, Shigeru Miyagawa

**Affiliations:** 1 Department of Cardiovascular Surgery, Osaka University Graduate School of Medicine, Suita, Osaka 565-0871, Japan; 2 Fujifilm Corporation, Ashigarakami 258-8577, Kanagawa, Japan; 3 Cardiovascular Division, Osaka Police Hospital, Tennoji 543-0035, Osaka, Japan; 4 Department of Cardiovascular Medicine, Osaka University Graduate School of Medicine, Suita 565-0871, Osaka, Japan; 5 Department of Medical Therapeutics for Heart Failure, Osaka University Graduate School of Medicine, Suita 565-0871, Osaka, Japan; 6 Department of Physiology, Osaka Dental University, 8-1 Kuzuha Hanazono-cho, Hirakata 573-1121, Osaka, Japan; 7 Laboratory of Human Immunology (Single Cell Genomics), WPI Immunology Research Center, Osaka University, Osaka, Japan; 8 Genome Information Research Center, Research Institute for Microbial Diseases, Osaka University, Osaka, Japan; 9 Department of Immunology and Molecular Medicine, Graduate School of Medicine, Osaka University, Suita 565-0871, Osaka, Japan; 10 Department of Future Medicine, Division of Health Science, Osaka University Graduate School of Medicine, Suita, Osaka 565-0871, Japan

**Keywords:** Bioengineering, Biotechnology, Cardiovascular medicine, Stem cells research

## Abstract

Human iPSC-derived cardiomyocytes (hiPSC-CMs) exhibit functional immaturity, potentially impacting their suitability for assessing drug proarrhythmic potential. We previously devised a traveling wave (TW) system to promote maturation in 3D cardiac tissue. To align with current drug assessment paradigms (CiPA and JiCSA), necessitating a 2D monolayer cardiac tissue, we integrated the TW system with a multi-electrode array. This gave rise to a hiPSC-derived closed-loop cardiac tissue (iCT), enabling spontaneous TW initiation and swift pacing of cardiomyocytes from various cell lines. The TW-paced cardiomyocytes demonstrated heightened sarcomeric and functional maturation, exhibiting enhanced response to isoproterenol. Moreover, these cells showcased diminished sensitivity to verapamil and maintained low arrhythmia rates with ranolazine—two drugs associated with a low risk of torsades de pointes (TdP). Notably, the TW group displayed increased arrhythmia rates with high and intermediate risk TdP drugs (quinidine and pimozide), underscoring the potential utility of this system in drug assessment applications.

## Highlights

• Traveling wave (TW) spontaneously originated in a close-loop cardiac tissue
• Rapid TW pacing enhanced the maturation of human iPSC-derived cardiomyocytes
• TW group showed improved response to drugs with various torsades de pointes risk

## Graphical abstract

**Figure:**
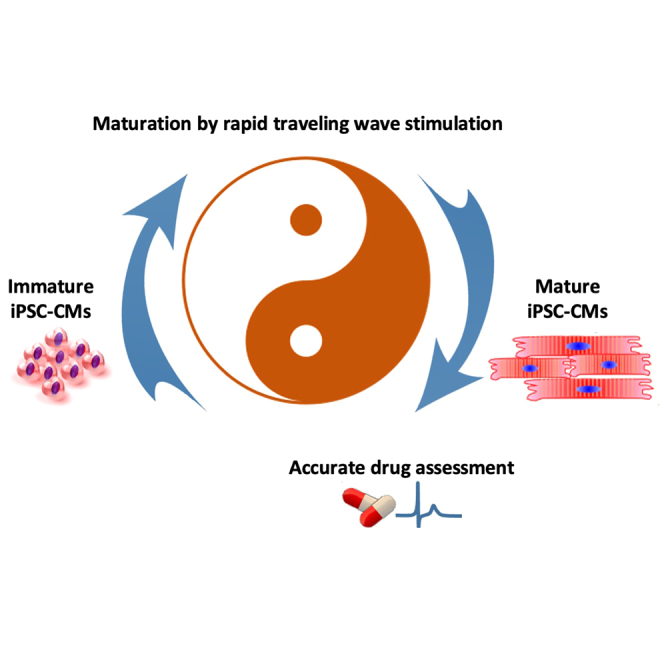

## Introduction

A precise evaluation of the safety and efficacy of newly developed medications is of vital importance for drug discovery. As a promising candidate for drug assessment, human iPSC-derived cardiomyocytes (hiPSC-CMs) have been extensively discussed for the development of a predictable *in vitro* drug cardiotoxicity screening assay^1^^,^^2^^,^^3^; the utility of hiPSC-CMs in detecting drug-induced proarrhythmic effects and their potential to evolve a new paradigm for *in vitro* proarrhythmic assays were previously demonstrated.^3^ However, iPSC-CM-created tissues exhibit significant differences in cellular features, such as morphology, contractility, and electrophysiology,^4^ when compared to native cardiac tissue, which could lead to variations in drug response and a higher susceptibility to arrhythmia.^1^^,^^3^

Currently, multiple approaches exist to induce the functional and morphological maturation of hiPSC-CMs. These include: (1) co-culture of hiPSC-CMs with other non-cardiomyocytes, such as fibroblasts and/or endothelial cells^5^^,^^6^^,^^7^ or mesenchymal stem cells^8^^,^^9^; (2) long-term culture^10^^,^^11^^,^^12^; (3) addition of small molecular or other soluble factors^13^^,^^14^^,^^15^; (4) using three-dimensional engineered tissue^16^^,^^17^^,^^18^^,^^19^^,^^20^^,^^21^^,^^22^^,^^23^; (5) using patterned or soft scaffold^24^^,^^25^^,^^26^; and (6) electrical or mechanical stimulation.^27^^,^^28^^,^^29^ These maturation methodologies can significantly improve the maturation of hiPSC-CMs in terms of sarcomere structure, calcium-handling properties, and electrophysiology, which dramatically affects the response of the cells to drugs. Specifically, long-term cultured hiPSC-CMs^10^ and three-dimensionally engineered tissues^22^ demonstrated less sensitivity to the calcium and hERG blocker verapamil. Electrical stimulation of mature cardiac tissue showed a physiologically relevant response to the beta agonist isoproterenol.^28^ hiPSC-CM tissues cultured on soft scaffolds showed a modest response to the IKr blocker E4031, which resembled the response of the adult myocardium.^26^

We previously developed a spontaneously originating, traveling wave-based platform capable of rapidly pacing and promoting the maturation of three-dimensional hiPSC-CM tissue rings.^30^^,^^31^ The pacing by traveling wave avoided the side effects of electrical stimulation such as heavy metal poisoning, electrolysis, pH shift, and the generation of reactive oxygen species (ROS)^16^^,^^32^ and there is no need for an external power supply. The tissue rings demonstrated upregulated cardiac-specific gene expression and enhanced oxygen consumption rate and contractility.^30^^,^^31^ In the present study, in order to adapt to the 2D monolayer cardiac tissue on a microelectrode array (MEA) as utilized in paradigms such as CiPA (FDA) and JiCSA (Japan iPS Cardiac Safety Assessment),^3^^,^^33^ we integrated the hiPSC-derived closed-loop cardiac tissue (iCT) with an MEA system for drug evaluation. Traveling waves (TWs) could spontaneously originate in the iCT and be maintained for >14 days, similar to those occurring in 3D tissue rings. The TWs promoted the maturation of hiPSC-CMs within the iCTs, showing improvements in conduction, ultrastructure, energetics, and contraction. When used for drug evaluation, compared to the tissues without TW (control group), the TW group showed an enhanced response to the β-adrenoceptor agonist isoproterenol, which corresponded to those recorded in a previous clinical study.^28^ In addition, ranolazine, a sodium and hERG blocker with a low risk of torsades de pointes (TdP),^3^ caused decreased arrhythmia and cessation episodes in the TW group than in the control group. Moreover, the TW group showed low data variation and less sensitivity to verapamil, a calcium blocker and hERG blocker, similar to previous reports on drug assessments with mature hiPSC-CMs.^22^^,^^34^ These results indicate that TW-matured iCT has potential for use in drug assessment.

## Results

### Traveling waves rapidly paced human induced pluripotent stem cell-derived cardiomyocytes-derived closed-loop cardiac tissue

**Figure 1:**
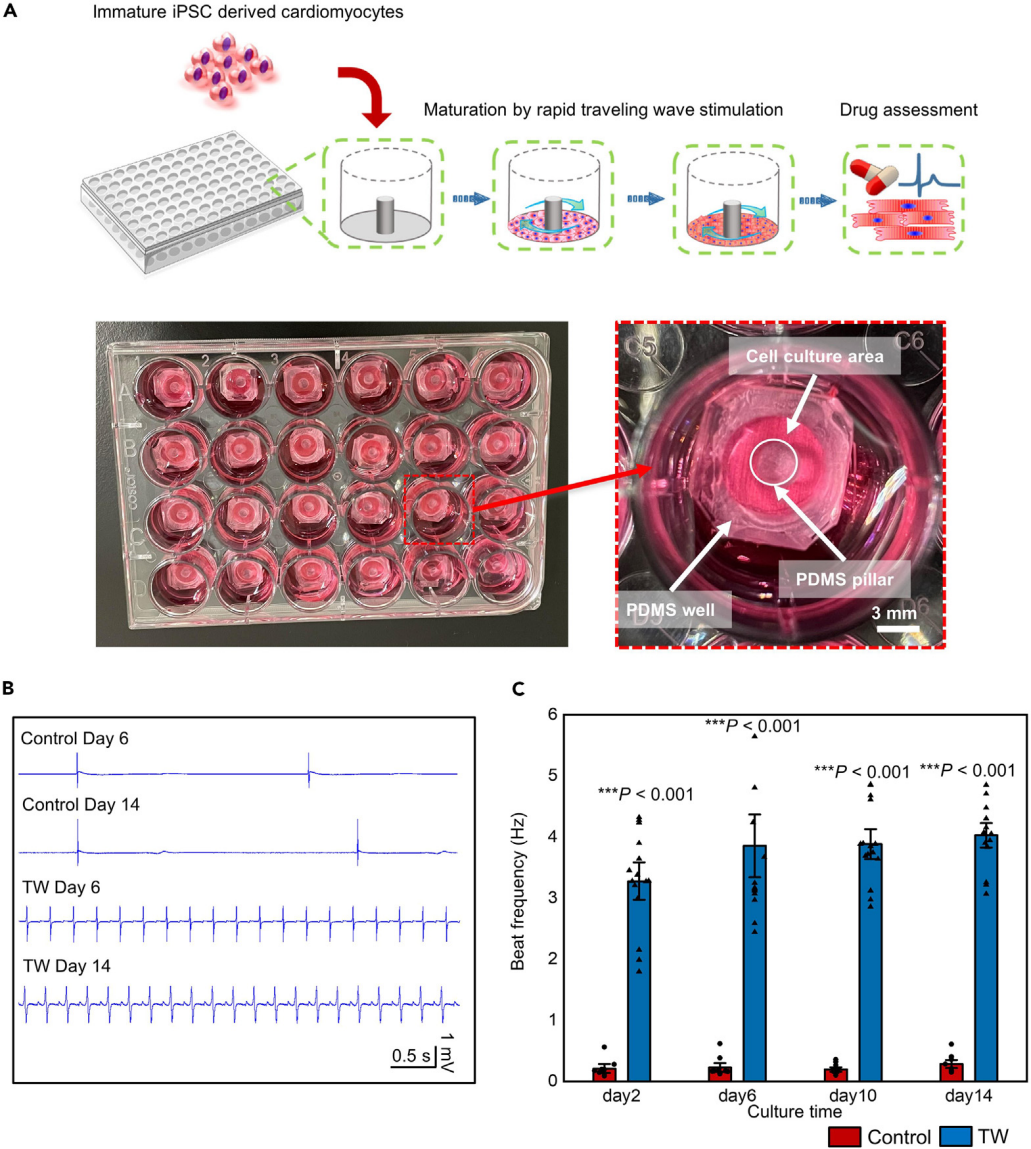
Traveling Waves (TWs) rapidly paced the cardiomyocytes within the closed-loop route (A) Schematic and image describing the cell plating and TW origination in the device. (B) MEA field potential signal of the iCTs with or without TW on day 6 and day 14. (C) Beat rates of iCTs at different culture times (Mean ± SEM; Control: n = 15; TW: n = 18 biologically independent samples from two differentiations). ∗∗∗p *<* 0.001 (Student’s *t* test).

The human fetal heart beats at ∼180 bpm, whereas the adult heart beats at ∼60 bpm.^35^ Electrical stimulation was shown to promote hiPSC-CM maturation.^28^^,^^29^ As a supplement, we previously developed a spontaneously originating TW system to pace CMs within 3D cardiac tissues.^31^ Using a protocol modified from our previous study, we created hiPSC-derived closed-loop monolayer cardiac tissue (iCT) by plating hiPSC-CMs in a PDMS well with a pillar in the center (Figure 1A; Figure S1). The looped TW propagated in the closed-loop monolayer tissue in 83.33 ± 13.6% of the samples (18 samples from three independent differentiations). The TW could pace the CMs within to beat at a much higher frequency (Figures 1B and 1C, Videos S1, S2, and S3) compared with the tissue without a traveling wave (TW: 220 ± 74 bpm vs. Control: 32.4 ± 30.1 bpm, n = 18, p < 0.0001, at day 6). The traveling waves were maintained for 14 days before being used for further assays, and they persisted for more than 100 days.^31^

Video S1. The hiPSC-derived closed-loop cardiac tissue (iCT) with traveling wave (TW) on day 6. The GCaMP3-253G1 CMs is used for demonstrating TW propagating within the tissue, related to Figure 1

Video S2. The hiPSC-derived closed-loop cardiac tissue (iCT) without TW (control group) on day 6, related to Figure 1

Video S3. The hiPSC-derived closed-loop cardiac tissue (iCT) with TW (TW group) on day 6, related to Figure 1

### Traveling waves regulate cardiac-related gene expression

**Figure 2:**
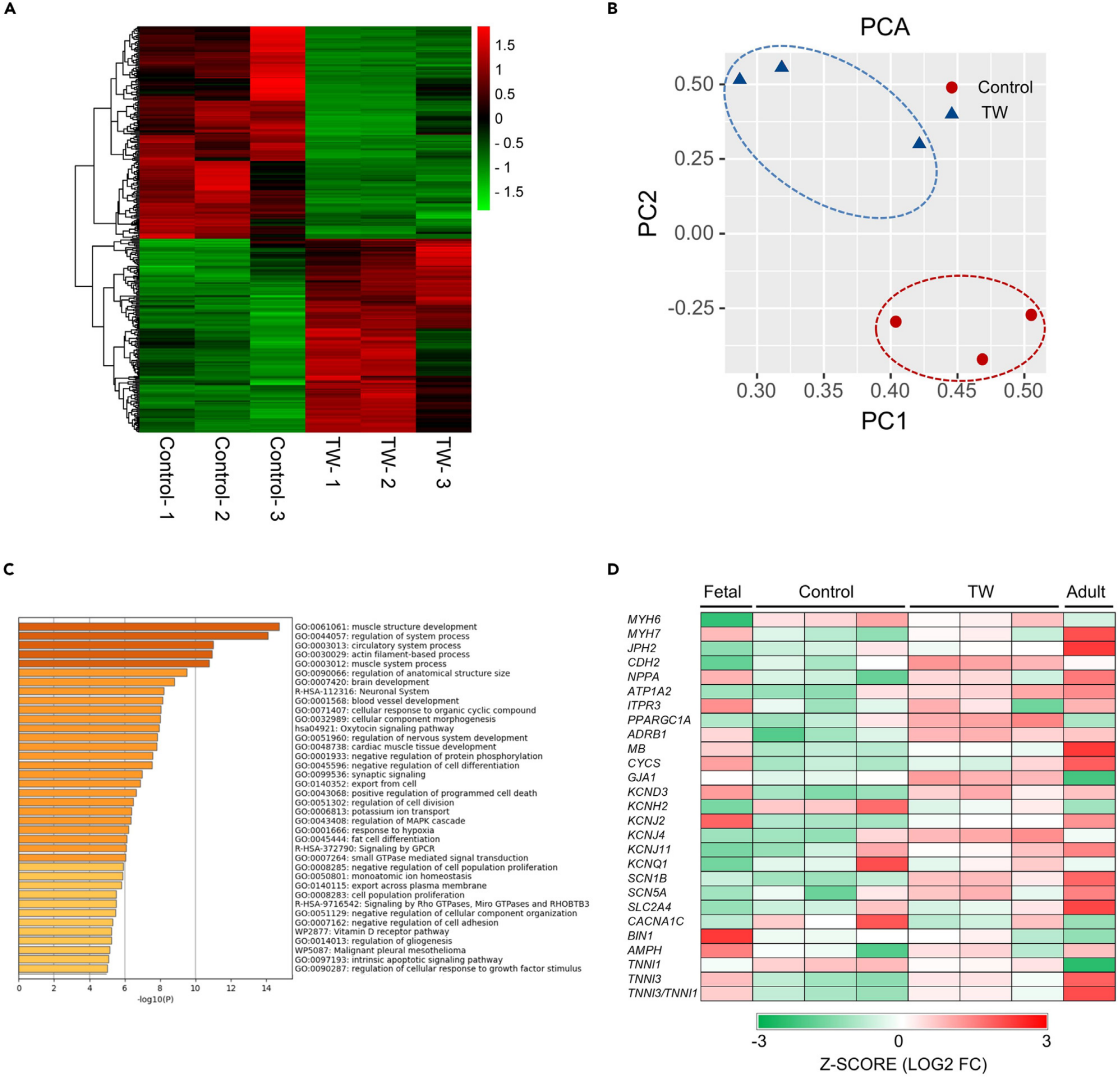
The transcriptome data indicated that TWs enhanced the expression of cardiac-related markers (A) Heatmap showing the hierarchical clustering of the correlation matrix resulting from comparison of expression values for each group. (B) Principal components analysis (PCA) of iCTs with or without TW based on RNA-sequencing data. (C) The 943 upregulated genes out of 26255 genes (adjusted p < 0.05; fold change >1.5) from the TW group (compared with the control group) were used for GO category analysis. The enriched terms are listed. (D) Heatmaps showing the expression of cardiac maturation-specific genes. To compare the gene expression in the TW and control group with fetal and adult heart data from a previous report, the FPKM data were converted into Transcripts Per Million (TPM).

To investigate how TWs regulate gene expression in iCTs, we used RNA sequencing to compare the gene expression profiles among different groups. The hierarchical clustering of the Spearman’s correlation heatmap (Figure 2A) and the principal components analysis (PCA; Figure 2B) indicated that there were closer correlations between iCTs without TW training (Control) and compared with iCTs with TW training. Next, we performed gene ontology (GO) analysis of the groups with and without TW. There were 943 upregulated genes out of a total of 26255 genes (adjusted p < 0.05; fold change >1.5) in the TW group compared with the control group. Several GO-enriched terms were associated with maturation, such as muscle structure development, actin filament-based processes, muscle system processes, and cardiac muscle tissue development (Figures 2C; Figures S2 and S3). The TW group (Figure 2D; Figure S2) showed regulated expression of genes associated with conduction (increased *GJA1, KCND3, KCNJ2, KCNJ4, KCNJ11, SCN1B*, and *SCN5A* expression), ultrastructure (increased *MYH7, JPH2, CDH2, TNNI3* expression and decreased *MYH6, TNNI1* expression), energetics (increased *NPPA, PPARGC1A, CYCS, MB,* and *ADRB1* expression), and calcium handling (increased *AMPH* and *ATP1A2* expression). The RNA-sequencing data were also compared with those of fetal and adult heart cells in a previous report.^36^ Compared with the control group, the TW group showed a change toward adult heart cells (Figure 2D).

The ion channel-related genes are of great importance for studying the drug response of hiPSC-CMs, we found the enhanced expression of several potassium ion channels (*KCND3*, *KCNJ2,* and *KCNJ4*) and sodium ion channels (*SCN1B* and *SCN5A*) in the TW group (Figure 2D; Figure S2). In addition, as mentioned in a previous report^37^ by the FDA on evaluating the usability of iPSC-CMs for drug assessment, the hiPSC-CM tended to have a higher expression of the calcium ion channel (*CACNA1C*) compared with those in the adults. We found decreased the expression of *CACNA1C* in the TW group (Figure 2D), which is closer to the adult group. Similarly, in the commercial Cor.4U, *KCNH2* also showed higher expression compared to that in the adult group.^37^ In the present work, the TW group showed reduced expression of *KCNH2*, which is closer to the levels in the adult group (Figure 2D) than those in the control group.

**Figure 3:**
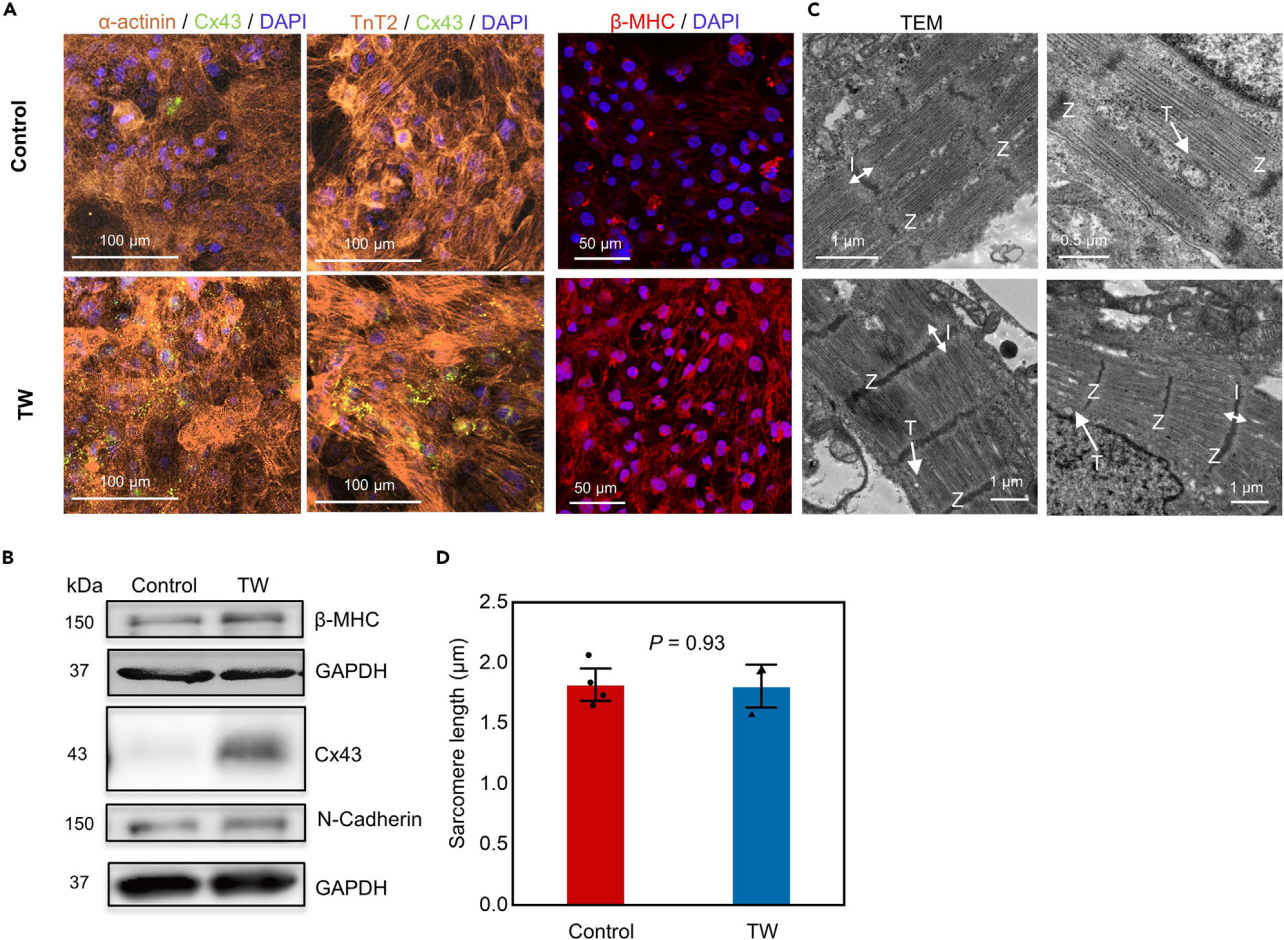
Traveling waves (TW) enhanced sarcomere maturation (A) Representative confocal images of iCTs with or without TW on day 14. Cardiomyocytes were stained with anti-α-actinin, anti-TnT2, anti-Cx43, anti-β-MHC, and DAPI. (B) Whole-cell lysates were extracted from TW and control groups and analyzed by western blotting using the indicated antibodies. (C) TEM analysis of iCTs from both the TW and control groups on day 14. Z: Z lines; I: I band; T: T-tubules. (D) The sarcomere length. Sarcomere lengths of CMs in both groups on day 14. (Mean ± SEM; Control: n = 4; TW: n = 3 biologically independent samples from two differentiations).

Next, we performed immunostaining for cardiac-specific markers; the TW group showed a significantly increased expression of the gap junction marker Cx43 (encoded by *GJA1*) and β-myosin heavy chain (β-MHC, encoded by *MYH7*), a cardiac maturity marker associated with muscle contraction (Figures 3A and 3B). Moreover, electron microscopy indicated that the CMs in the TW group had larger sarcomere bundles and well-defined Z disks, I-bands, T-tubules, and myofibrils than those in the control group (Figure 3C). There is no significant difference in the sarcomere length between both groups (Figure 3D). We then confirmed the high expression of proteins such as β-MHC (encoded by *MYH7*), Cx43 (encoded by *GJA1*), and N-cadherin (encoded by *CDH2*) in the TW group by western blotting (Figure 3B). These data indicated that the CMs in the TW group were more mature than those in the control group in terms of sarcomere structure, gap junction, and other maturation-related marker expression.

### Traveling waves improved the functional maturation of CMs

**Figure 4:**
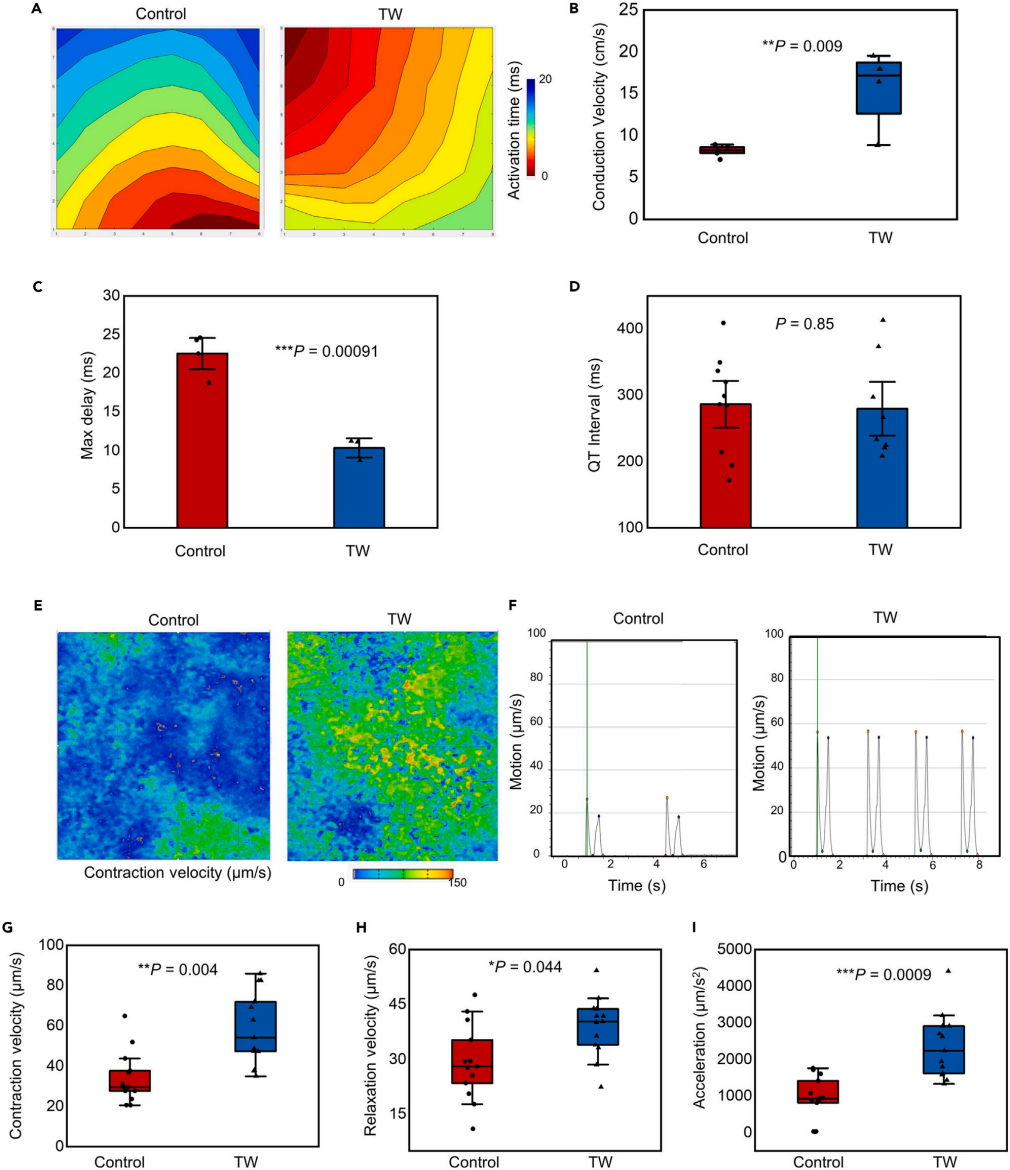
Traveling waves (TWs) improved the electrical conduction and the contractility of hiPSC-derived close-loop cardiac tissue (iCT) (A) Activation maps showing the propagation of contractility on day 14, the TWs have been removed before recording to allow spontaneous beating. Each of the points on the axis represent an electrode (8 × 8, spacing 200 μm) on MEA. (B–D) The conduction velocity (B), Max delay (C) and QTinterval (D) of the contraction of both groups (Mean ± SEM; Conduction velocity: Control: n = 5; TW: n = 4 biologically independent samples from three differentiations; Max delay: Control: n = 4; TW: n = 3 biologically independent samples from two differentiations; QTinterval: Control: n = 10; TW: n = 8 independent biologically samples from four differentiations. ∗∗p < 0.01, ∗∗∗p < 0.001 (Student’s *t* test). (E) Representative velocity image of the control and TW groups using a motion analysis system. Red and blue represent high and low velocities, respectively. (F) Plot of a motion waveform showing contraction and relaxation velocity peaks. The green line marks the time point of the data in (e). (G–I) Contractile properties of the Control group and TW group. (g) Contraction velocity, (h) relaxation velocity, and (i) acceleration (Mean ± SEM; Control: n = 10; TW: n = 13 biologically independent samples from three differentiations). ∗p *<* 0.05, ∗∗p *<* 0.01, ∗∗∗p *<* 0.001 (Student’s *t* test).

To evaluate the functional maturation of CMs, we performed electrophysiology recording and motion analysis on the two groups. Both the TW and control groups showed the homogeneous propagation of contractions; however, the conduction velocity of the TW group was significantly higher than that of the control group, while the Max delay of the TW group was smaller than that of the control group (Figures 4A–4D), which agrees well with the enhanced expression of the gap junction gene *GJA1* (Figure 2D) and the protein Cx43 (Figure 3) in the TW group. Both the control and TW groups demonstrated QT intervals at approximately 300 ms, which is much shorter than those (∼600 ms) of the LQT2 patient.^38^ Furthermore, the motion analysis revealed that, after training with TW, the CMs in the TW group showed significantly higher contractility than those in the control group (Figures 4C–4I), including higher contraction velocity (58.68 ± 17.49 μm/s vs. 36.74 ± 12.91 μm/s; p = 0.004), relaxation velocity (39 ± 7.87 μm/s vs. 30.19 ± 10.96 μm/s; p = 0.044), as well as acceleration (2359.54 ± 844.68 μm/s vs. 1200.29 ± 361.64 μm/s^2^; p = 0.0008).

**Figure 5:**
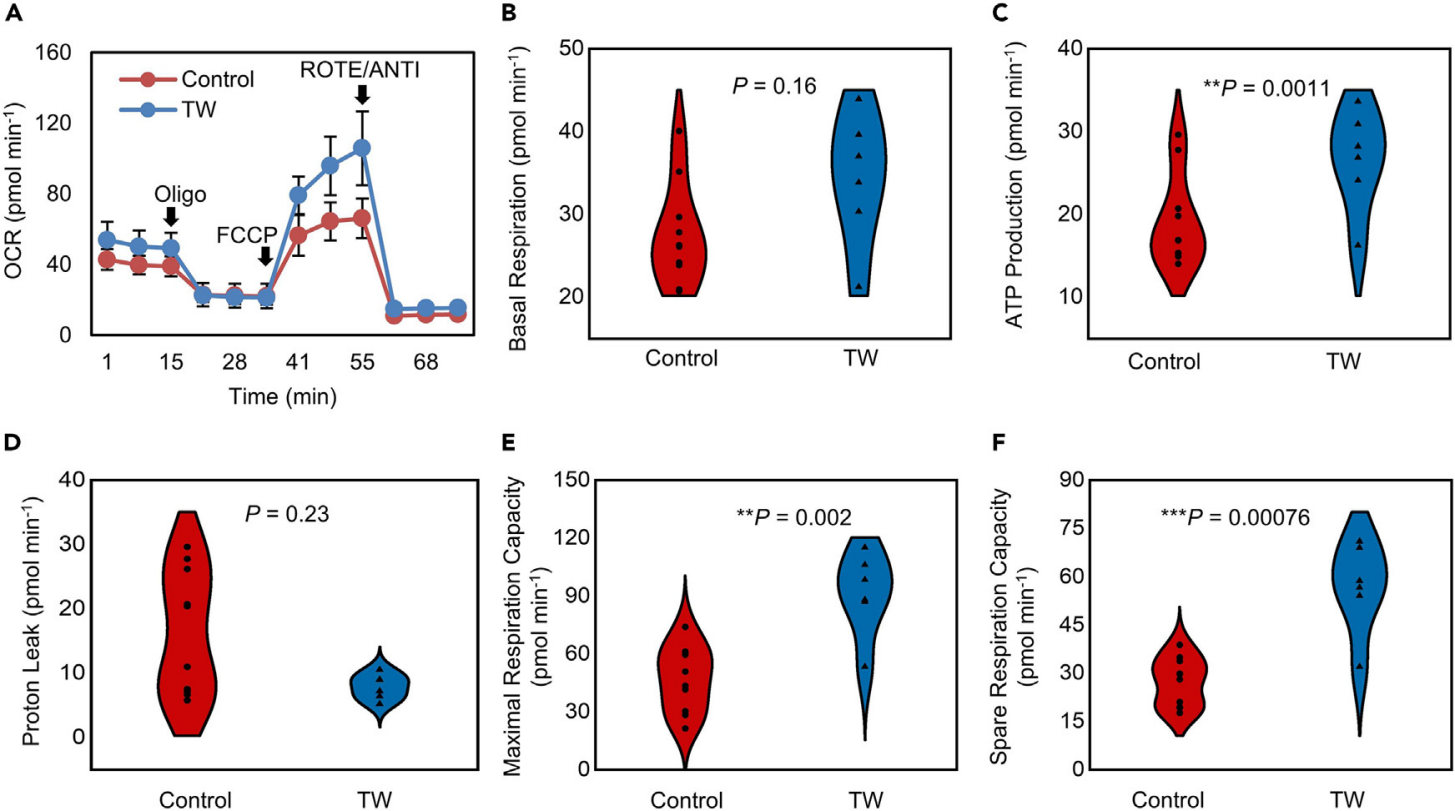
Traveling Waves (TW) improved the mitochondrial function of hiPSC-derived closed-loop cardiac tissue (iCT) (A) Results of oxygen consumption rate (OCR) assays. Oligo, oligomycin; FCCP, carbonyl cyanide-4-(trifluoromethoxy) phenylhydrazone; ROTE/ANTI, rotenone and antimycin A. (B–F) Quantification of the basal respiration (b), ATP production (c), proton leak (d), maximal respiration capacity (e), and spare capacity (f) (Control: n = 7; TW: n = 6 biologically independent samples from one differentiation). ∗p *<* 0.05, ∗∗p *<* 0.01, ∗∗∗p *<* 0.001 (Student’s *t* test).

An extracellular flux analyzer was then used to evaluate the mitochondrial function of the CMs in both groups. Mitochondrial ATP synthesis was inhibited by the addition of oligomycin. A proton-gradient discharger (FCCP) was added to evaluate the maximum mitochondrial respiration (Figures 5A–5F). There was no significant difference in the basal respiration (27.96 ± 6.41 pmol min^−1^ vs. 34.24 ± 7.28 pmol min^−1^; p = 0.16) and proton leak levels (7.68 ± 1.79 pmol min^−1^ vs. 11.94 ± 7.44 pmol min^−1^; p = 0.23) between the two groups. The TW group showed higher ATP production (26.56 ± 5.56 pmol min^−1^ vs. 16.02 ± 1.76 pmol min^−1^; p = 0.0011), maximum respiration rate (90.89 ± 19.68 pmol min^−1^ vs. 55.23 ± 10.54 pmol min^−1^; p = 0.0029), and spare capacity (56.65 ± 12.81 pmol min^−1^ vs. 27.27 ± 8.12 pmol min^−1^; p = 0.00076) than the control group, indicating enhanced mitochondrial activity.

### Traveling waves trained human induced pluripotent stem cell-CMs for drug assessment

**Figure 6:**
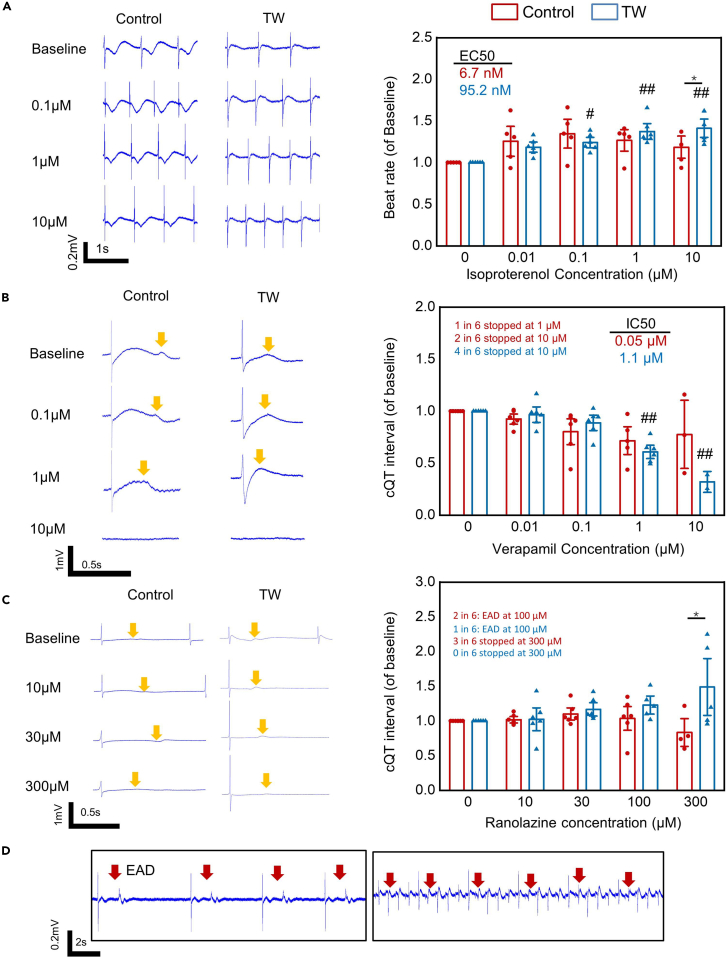
Low TdP risk drug response of hiPSC-derived closed-loop cardiac tissue (iCT) with or without traveling wave (TW) training (A–C) Representative trace (left) and drug effect (right) of CMs (253G1) treated with isoproterenol (β adrenoceptor agonist), verapamil (calcium blocker, low TdP Risk), and ranolazine (sodium and hERG blocker, low TdP risk) (Mean ± SEM; isoproterenol: Control: n = 5; TW: n = 6; verapamil: Control: n = 6; TW: n = 6; ranolazine: Control: n = 6; TW: n = 6; independent biologically samples from three differentiations). ∗p *<* 0.05 (Student’s *t* test); #p *<* 0.05, ##p *<* 0.01 vs. values before drug (ANOVA). (D) Representative trace of early afterdepolarization (EAD), the red arrows mark the EAD. The yellow arrows mark the Twave.

**Figure 7:**
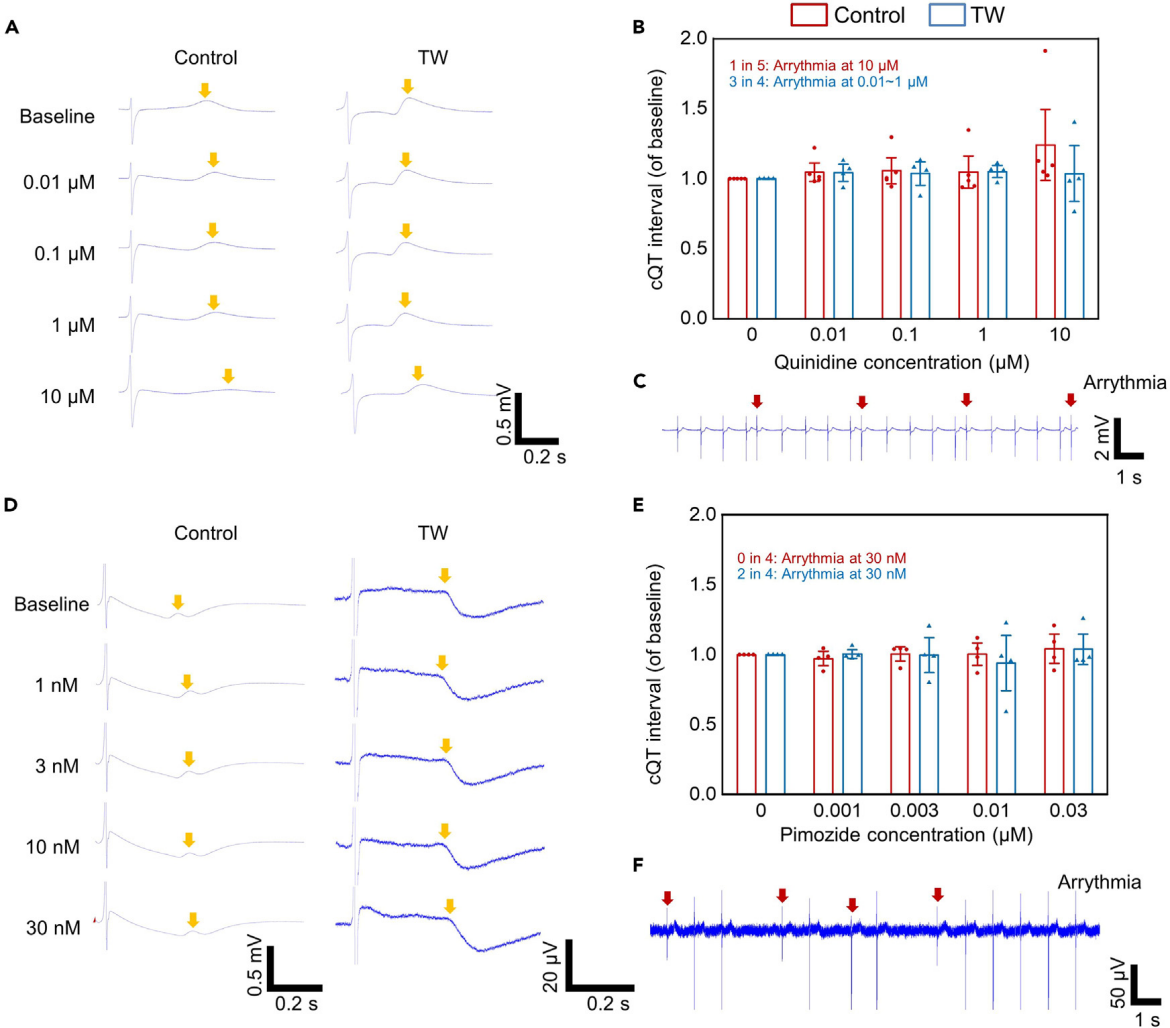
High/intermediate TdP risk drug response of hiPSC-derived closed-loop cardiac tissue (iCT) with or without traveling wave (TW) training (A–F) Representative trace (A), drug effect (B), arrythmia-like events (C) of CMs (253G1) treated with quinidine (high TdP risk). Representative trace (D), drug effect (E), arrythmia-like events (F) of CMs (253G1) treated with pimozide (intermediate TdP risk). The red arrows indicate the arrythmia-like events. The yellow arrows mark the Twave. (Mean ± SEM; quinidine: Control: n = 5; TW: n = 4; pimozide: Control: n = 4; TW: n = 4; biologically independent samples from two differentiations).

We further evaluated the drug response of iCT with two types of cardiomyocytes (253G1-derived cardiomycytes and commercially available iCell^2^). Rather than directly culturing iCTs on expensive MEA chips, hiPSC-CMs were cultured on the device with a layer of permeable fiber scaffold attached to the bottom. After culturing and training in a normal 24-well plate (Figure 1A), the iCT was directly transferred onto an MEA for drug tests (Figures S4A and S4B), as well as for culturing after the drug tests. Moreover, the MEA chips could be immediately reused for the next iCT recording after washup. The TW-paced iCT beat significantly faster than the control group (Figures S4C and S4D) and showed enhanced sarcomere structure (Figure S4E), which is similar to the results obtained after using the device without a fiber scaffold. The iCTs were then used for drug response tests (Figures 6 and 7; Table 1; Figures S5–S7), including adrenoreceptor agonist (isoproterenol), sodium/potassium current blocker (ranolazine), calcium blocker (verapamil), sodium channel blocker (mexiletine), potassium blocker (E4031), and a negative control (aspirin). Drugs with high (quinidine) and intermediate (pimozide) torsades de pointes (TdP) risks were also assessed to evaluate the system.

**Table 1:** The drugs being assessed and the arrhythmia related events occurred post drug administration

Drug name	Note	Proarrhythmia risk	Arrhythmia-related events (253G1)	Arrhythmia-related events (iCell^2^)
isoproterenol	β adrenoceptor agonist	Low	N/A	N/A
verapamil	calcium blocker	Low	Cessation in 3/6 (Control), 4/6 (TW)	Cessation in 0/6 (Control), 1/4 (TW)
ranolazine	sodium and hERG blocker	Low	EAD in 2/6 (Control) in 1/6 (TW), Cessation in 3/6 (Control), 0/6 (TW)	EAD in 3/8 (Control) in 1/9 (TW), Cessation in 2/8 (Control), 1/9 (TW)
E4031	potassium blocker	Low	N/A	N/A
mexiletine	sodium channel and hERG channel blocker	Low	Arrhythmic activities in 1/9 (Control and TW)	Arrhythmic activities in 2/8 (Control), 0/9 (TW)
pimozide	hERG blocker	Intermediate	Arrhythmic activities in 0/4 (Control), 2/4 (TW)	N/A
quinidine	hERG blocker	high	Arrhythmic activities in 1/5 (Control), 3/4 (TW)	N/A
aspirin	negative control	N/A	N/A	N/A

Upon exposure of iCT to β-adrenergic stimulation with isoproterenol, the trained tissue from the 253G1 line showed a chronotropic effect at all the tested concentrations (from 0 μM to 10 μM, 141.24% ± 16.43%, p < 0.001). The control group demonstrated a chronotropic effect only between 0 μM and 0.1 μM (134.76% ± 23.1%, p < 0.01) and the beat rate decreased between 0.1 μM and 10 μM (Figure 6A). The EC_50_ values of the TW and control groups were 95.2 nM and 6.7 nM, respectively, similar to those reported in a previous study on the electrical stimulation of the matured cardiac tissue.^28^ Identically, the iCT with TW made using iCell demonstrated a lower EC_50_ value but an enhanced reaction to isoproterenol at all tested concentrations (Figure S5). This result agrees well with that of a previous report^39^ in which the chemical factor-matured cardiac tissue showed a chronotropic effect between 0 μM and 10 μM, whereas the control group had a maximum beat rate between 0.01 and 0.1 μM. In addition, the improved expression of the β-adrenergic receptor (*ADRB1*) may be correlated with the improved reaction of iCT to β-adrenergic stimulation (Figure 2D).

We next evaluated the response of both groups from the 253G1 line-derived cardiomycytes to the calcium blocker verapamil (Figure 6B), which is also a hERG blocker with no reports of QT prolongation or TdP in humans.^40^ Both groups showed a shortened QT interval with the addition of verapamil; 4 out of 6 samples in the TW group stopped beating at 10 μM, while 1 and 2 out of 6 samples in the control group stopped beating at 1 and 10 μM, respectively, similar to a previous report.^10^ In addition, the TW group showed lower data variation than the control group. The IC_50_ value of the control group (0.05 μM) was close to the free effective therapeutic plasma concentration (ETPCunbound),^10^^,^^40^ while the IC_50_ of the TW trained group (1.1 μM) resulted in a higher safety margin (IC_50_/ETPCunbound) of 20, which is close to the safety margins (20–30^22^) typically required by pharmaceutical companies for the development of new drugs. This improvement was also achieved previously using long-term cultured iPSC-derived cardiac tissue^10^ and 3D engineered iPSC-derived cardiac tissue.^22^ In the iCell-made iCT (Figure S5), 1 in 4 TW samples stopped beating, and 0 of the 6 control samples stopped beating at a concentration of 10 μM. The IC_50_ of the two groups was 2.59 and 2.73 μM, respectively. The inter-cell line variation between 253G1-derived CM and iCell may contribute to the different responses to Verapamil.

The sodium blocker ranolazine is a known hERG blocker that prolongs the QT interval, but has a low risk of TdP. It was previously reported that ranolazine causes early afterdepolarization (EAD) in hiPSC-CMs at clinically relevant concentrations (C_max_, 2–6 μM).^1^ Another multisite study showed that arrhythmia or cessation could occur in approximately 40% of samples at 100 μM (>50-fold C_max_).^3^ In the present study, we observed EAD in 2 out of 6 control samples and 1 out of 6 TW samples at 100 μM. In addition, the control group showed a downward trend in the cQT interval at concentrations beyond 30 μM, while 2 out of 6 samples showed cessation of beating at 300 μM. Meanwhile, the TW group showed a continuous increase in the cQT interval, and none of the 6 samples showed cessation of beating at 300 μM (Figure 6C). Similarly, in the iCT made by iCell, 3 in 8 control samples showed EAD at 100 μM, while 1 in 9 TW samples showed EAD (Figure S5C). When the drug concentration was increased to 300 μM, 2 in 8 control samples stopped beating compared to the 1 in 9 TW samples. The cessation or increase in arrhythmia in the control group could have resulted from the low maturity of the hiPSC-derived cardiomyocytes, as discussed in previous reports.^1^^,^^41^^,^^42^

The potassium blocker E4031 was also evaluated, and both the control and TW groups showed QT prolongation after E4031 treatment; no arrhythmic activities were observed in either group at all tested concentrations (Figures S6 and S7). Mexiletine, a sodium and hERG channel blocker, was also added to the two groups. QT prolongation was observed in both groups. In 253G1-derived cardiomycyte samples, arrhythmic activities occurred in one each of the control (nine samples) and TW samples (nine samples) at 100 μM (>40-fold C_max_^3^). In iCell samples, 2 of the 8 control samples showed arrhythmic activities, while none of the 9 TW samples showed abnormal activities. The negative control, aspirin, showed no significant difference between the two groups.

Since most of the above drugs have low or no TdP risk, high (quinidine) and intermediate (pimozide) TdP risk drugs were also applied to the iCT tissues (Figure 7; Figure S8). Quinidine is a known high TdP risk drug that could induce arrhythmia-like events at concentrations close to clinical C_max_ (3 μM).^3^ Both groups showed the prolongation of QT intervals after quinidine treatment. However, 1 in 5 control samples showed arrythmia-like events at 10 μM, while 3 in 4 TW samples showed arrythmia-like events at a lower concentration (1 sample each at 0.01, 0.1, and 1 μM, respectively). In addition, we applied the intermediate risk drug pimozide to the iCTs. While 4 control group tissues showed no arrythmia-like events at 30 nM, a concentration 70-fold higher than C_max_ (0.4 nM), 2 out of 4 TW group tissues showed arrythmia-like events at 30 nM. These results indicated that the TW group may have higher sensitivity to the two types of high- and intermediate-risk TdP drugs.

### Integrin related pathway plays a role in regulating iCT maturation

**Figure 8:**
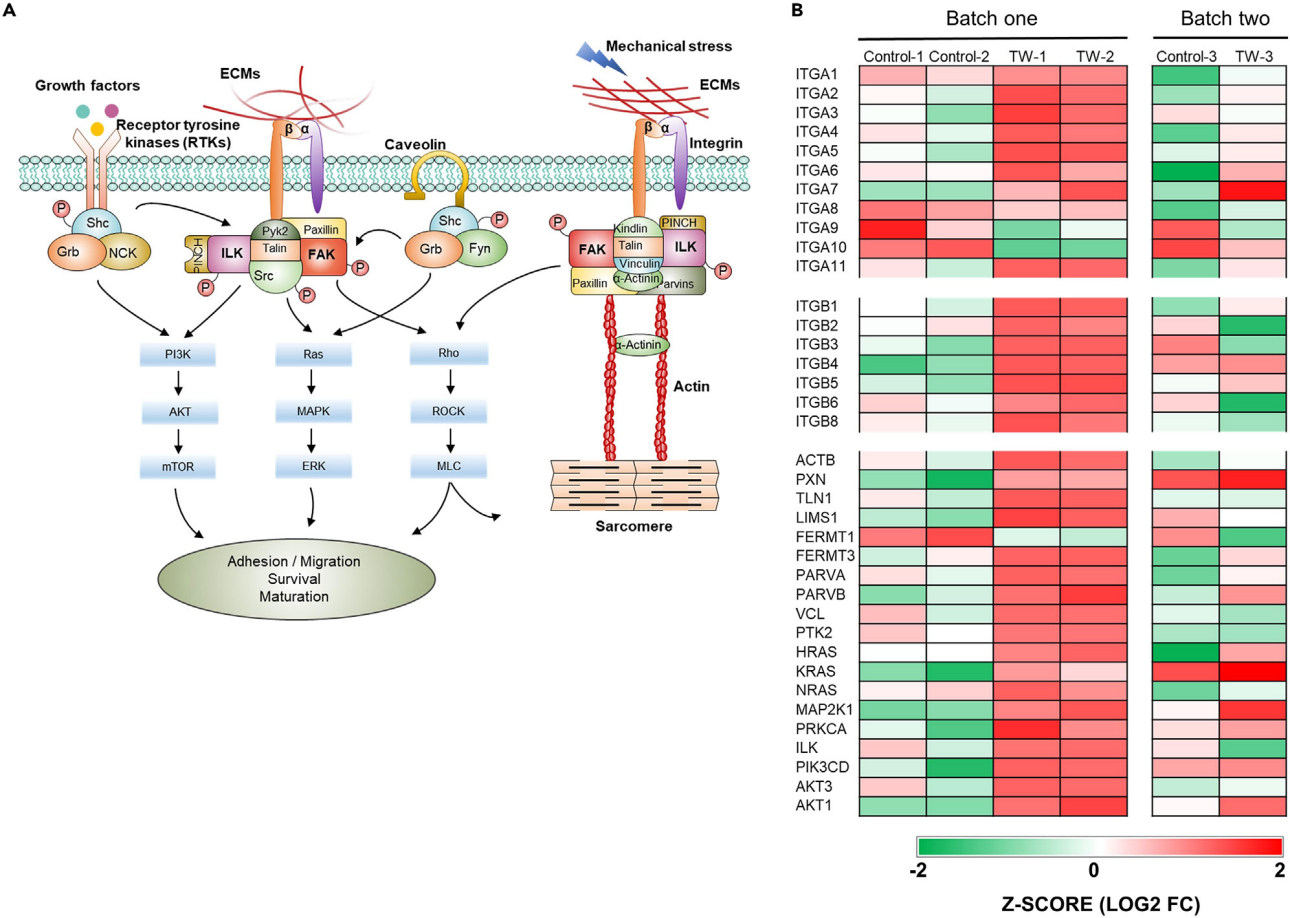
Proposed model for the traveling wave (TW)-induced hiPSC-derived closed-loop cardiac tissue (iCT) maturation (A) Schematic representation of an integrin pathway that could lead to the survival, adhesion/migration and growth/maturation of the cells. (B) Heatmaps showing expression of ECM-related genes. The data were collected from three biologically independent samples from two differentiations (batches).

Integrins are a family of cell adhesion receptors that bind to the extracellular matrix, cell-surface, and soluble ligands.^43^^,^^44^ They are especially important for cell-extracellular matrix (ECM) adhesion, structural organization, and transducing mechanical signals from the ECM into cardiomyocytes.^26^^,^^45^^,^^46^ Β1 integrins are abundant in the adult heart and play an important role in the hypertrophic response/maturation of ventricular myocytes.^46^^,^^47^^,^^48^ The soft Matrigel substrate has been reported to induce the maturation of hiPSC-CM monolayers, with integrin signaling playing a vital role.^26^ We hypothesized that the integrin-related pathway mediated the traveling wave-induced maturation of hiPSC-CMs (Figure 8A). RNA-sequencing data indicated that most of the alpha (*ITGA*) and part of the beta (*ITGB*) subunits of integrin in the TW group showed marked upregulation compared with the control and day 0 values (Figure 8B). Similar to that in a previous report,^26^ a number of downstream genes were also remarkably upregulated; these included ECM-related genes, actin (*ACTB*) and parvin (*PARVA* and *PARVB*), and genes related to integrin subtype-specific activation of pro-survival and pro-maturation signaling pathways, such as Ras (*HRAS*, *NRAS*), PI3K (*PIK3CD*), Akt (*AKT1 and AKT3*), and ERK (*MAP2K1*). These findings indicate that TWs promoted hiPSC-CM maturation through the integrin-related signaling pathway.

## Discussion

Rapid pacing of hiPSC-CMs is proven to be effective for their maturation.^28^^,^^29^ We previously developed a spontaneously originating TW for rapid pacing and maturation of hiPSC-CMs in a three-dimensional tissue ring.^30^^,^^31^ To date, cardiac safety paradigms such as CiPA (FDA) and JiCSA (Japan iPS Cardiac Safety Assessment) utilize 2D monolayer cardiomyocytes cultured on MEA systems for drug evaluation.^3^^,^^33^ In the present study, we created hiPSC-derived closed-loop cardiac tissue (iCT) on a 2D substrate. TWs can spontaneously originate within the 2D sheet and rapidly pace hiPSC-CMs. Similar to that observed in the 3D tissue ring, TWs promoted the maturation of hiPSC-CMs within the monolayer tissue, which showed improvement in multiple features such as conduction, ultrastructure, energy, and contraction.

Interestingly, the TW-trained iCT demonstrated improved oxygen consumption rate, as well as survival rate after hypoxic culture, compared with the control group. This is probably because the high expression of respiration-related proteins, myoglobin, and cytochrome *c* in the TW group enhanced the oxygen flux,^49^ mitochondrial function, and ATP synthesis under hypoxic conditions and promoted the survival of cardiomyocytes. In future studies, it would be interesting to further analyze the survival capability of TW-trained CMs after transplantation in animal models of myocardial infarction.

It has long been hypothesized that mature hiPSC-CM tissue could resemble adult myocardium and would be more suitable for the evaluation of drug response. There have been reports on the assessment of the response of cardiac tissue with improved maturation to different drugs: (1) Rapid pacing mature hiPSC-CMs showed physiological responses to isoproterenol^28^; (2) hiPSC-CMs matured by long-term culture^34^ or engineered into aligned 3D μ-tissues^22^ demonstrated less sensitivity to verapamil, an L-type calcium channel blocker that has a potent effect on hERG; and (3) cardiac tissue matured by a soft substrate showed modest response to the Ikr blocker E4031, which resembled the response of adult myocardium.^26^ In our study, TW-matured hiPSC-CMs showed similar responses to several of the previously mentioned drug types, such as isoproterenol and verapamil, but not to E4031. In addition, the TW group showed an improved response to the sodium blocker ranolazine, which is a known hERG blocker that prolongs the QT interval but has a low risk of TdP. The cessation or increased arrhythmia in the control group could be due to the immaturity of hiPSC-CMs, as discussed in previous reports.^1^^,^^41^^,^^42^ In addition, as indicated by the RNAseq data, the gene expression level of multiple ion channels and beta receptors is regulated toward adult levels in the TW group, compared with that of the control group. This may be related to the improved response to drugs such as beta agonists, calcium blockers, and hERG blockers. In the future, more efforts are needed to investigate the underlining mechanisms and correlation between the ion channel maturation and the drug response.

Recently, several multisite multiline studies have been performed to prove the capability of hiPSC-CMs as an *in vitro* proarrhythmia model.^3^^,^^33^ The results validated the utility of hiPSC-CMs in predicting drug-induced proarrhythmic effects as part of an evolving paradigm. To further validate the utility of TW-induced iCT as an enhanced drug assessment candidate, our future work will also include site-to-site and line-to-line variation evaluation.

Since the TW is a spontaneous activity that could not be precisely controlled and there are still significant sample-to-sample variations in data such as conduction velocity and transcriptome data. More efforts are needed to investigate the most efficient way to control the origination, maintaince, and observation of TW: For example, the TW tissue could be prepared with the optogenetic cell line that could be paced by light stimulation^50^ for controllable origination of TW. Moreover, the cell line with Ca^2+^ indicator^51^ may be used for improved observation of TW activity such as propagation velocity and the beating frequency. In addition, TW samples maybe categorized by the frequency range, which allows further reducing the sample-to-sample variations.

Instead of pacing and maturing hiPSC-CMs before drug assessment, there are also reports on using electrical^52^ or optical stimulation^53^^,^^54^ to pace the hiPSC-CMs during drug assessment. The pacing could control the spontaneous beating of the hiPSC-CMs and thus reduce the variation caused by the beat rate. The electrical pacing sample showed reduced response to multiple cardiac ion channel blockers and enabled more accurate rate-dependent drug evaluation.^52^ However, the optically stimulated group was not found to be substantially improved with respect to proarrhythmic risk predictions compared with the non-paced group.^53^ In the present study, the TW was stopped before the hiPSC-CMs were used for drug assessment because the high frequency of the TW (3–4 Hz) may interfere with the CM response. Following this, the beat of iCT would dramatically decrease and stabilize at approximately 1 Hz, relative to the human heart rate, allowing for a more accurate drug response. The results on stimulation during drug test, together with those reports on stimulation before a drug test, indicate that the stimulation time and stimulation delivery method may play an important role in the use of hiPSC-CM in drug assessment.

In our previous experience, it was difficult to culture 2D cardiac tissue on MEA chips for longer terms (>14 days), because the CMs would peel off from the substrate, especially when the MEA chips were reused several times. Culturing the cells on a fiber layer would resolve the problem of attachment, while allowing the recording of the signal using electrodes below the fiber sheet. This would allow long-term (∼months) evaluation of the chronic response of cardiac tissue to drug candidates.^23^ In addition, because the cells do not need to attach to the surface of MEA chips, the chips could be reused in the same batch of tests by simply replacing the tissue with a new one. This can significantly reduce the use of expensive MEA chips. To improve throughput and reduce the cost of drug assessment, hiPSC-CM tissues have been made using as few as thousands of cells in 3D cluster microtissues.^7^^,^^55^ Although the cell number required for creating closed-loop cardiac tissue has been reduced to 2×10^5^ from 4 × 10e^5^ for the 3D rings,^31^ it is still not suitable for high-throughput assays. Further optimization is required to further scale down the closed-loop device and reduce the required cell number. In previous 3D ring formation, the sarcomere length of TW group has been improved, however, there is no similar improvement in 2D tissue, this may be caused by the different culture system between 2D and 3D system, as significant difference has been found between CM cultured and matured under 2D^56^^,^^57^ and 3D^58^ condition, in the future, we will further improve the TW device to adapted the 3D tissue while allowing the recording by MEA system. For example, the soft Matrigel could be used to coat the surface of scaffold to allow the 3D cells culture and sarcomere length improvement.^59^ Furthermore, it is important to note that the RNA-seq analysis in the present study is based on the assessment of average gene expression within the samples. Nonetheless, the differentiated CMs derived from hiPSCs frequently comprise a heterogeneous mixture of cell types, including atrial, ventricular cells, and various other subpopulations. In the future, the adoption of Single-cell RNA sequencing (scRNA-seq) holds the promise of providing more precise genetic information and enhanced resolution of maturation states for each of these subpopulations.” In addition, there may be variation between different batches of experiment, for example, the *GJA1* expression in the adult group, adapted from previous report^60^ is lower than those in TW and control group (Figure 2D), and the *GJA1* expression level of adult heart (Normalized to TPM) varies a lot among the different articles.^61^^,^^62^^,^^63^ In the future, it may be more appropriate to perform the comparison within the same batch to minimize the variation.

In the present design, the PDMS is used to fabricate the device, which may have the issue of compounds sticking,^64^^,^^65^ in future, the other material such as polystyrene will be used to replace the PDMS. Moreover, because cardiovascular liability of drugs commonly occurs via the altered function of the contractile myocardium, the contractility or contractile force has also been suggested as an evaluation factor for cardiac safety paradigms, in addition to the electrophysiology.^66^^,^^67^ Our future optimization might include integrating the evaluation of contractile force into the present device.

### Limitation of the study

The study is constrained by several limitations, including: a. The inability of the TW-paced hiPSC-CM to achieve a maximum frequency of 6 Hz, as reported in previous electrical stimulation studies.^68^ Furthermore, restarting TW after interruption is time-consuming and demands specialized skills. b. The digestion process applied to cardiac tissue has the potential to disrupt its tissue-specific properties. c. Utilizing multiple recording systems, such as calcium and/or voltage-based imaging, is necessary for obtaining a more comprehensive dataset than that of the MEA system. This enhancement not only facilitates the recording of tissue activity in the initial days (0–4 days) but also enables the mapping of activation in a larger area than the MEA system allows.

### Conclusion

We observed the TW phenomenon in a closed-loop monolayer cardiac tissue. TWs could pace the hiPSC-CMs to beat at a high frequency and maintain it for more than two weeks. The TW-paced hiPSC-CMs showed improved sarcomeric and functional maturation. According to the drug response data, the mature hiPSC-CMs demonstrated improved drug responses resembling those of the adult myocardium, holding potential as a candidate for drug discovery with improved robustness and accuracy.

## STAR★Methods

### Key resources table

**Table:** 

REAGENT or RESOURCE	SOURCE	IDENTIFIER
**Antibodies**		

Mouse monoclonal anti-α-actinin	Sigma-Aldrich	Cat#A7811; RRID: AB_476766
Mouse monoclonal anti-troponin T2 (TnT2)	Santa Cruz Biotechnology	Cat#SC-20025; RRID: AB_628403
Rabbit polyclonal anti-connexin 43	Sigma-Aldrich	Cat#C6219; AB_476857
Mouse monoclonal anti-β-MHC	Santa Cruz Biotechnology	Cat#SC-53089; RRID: AB_2147281
Alexa Fluor 488 anti-mouse IgG	Jackson Immuno Research	Cat#715-546-150; RRID: AB_2340849
Alexa Fluor 594 anti-mouse IgG	Jackson Immuno Research	Cat#715-586-150; RRID: AB_2340857
DyLight-594 anti-mouse IgM	Jackson Immuno Research	Cat#715-516-020; RRID: AB_2340843
Alexa Fluor 647 anti-rabbit IgG	ThermoFisher	Cat#A21245; RRID: AB_2535813
Alexa Fluor 488 anti-rabbit IgG	ThermoFisher	Cat#A21206; RRID: AB_2535792
mouse monoclonal IgG (isotype)	Santa cruz	Cat#sc-2025; RRID: AB_737182

**Chemicals, peptides, and recombinant proteins**		

4′,6-Diamidino-2-phenylindole, dihydrochloride (DAPI)	Wako Pure Chemical Industries, Ltd.	Cat#342-07431
Primate embryonic stem cell medium	ReproCELL	Cat#RCHEMD001
Human basic fibroblast growth factor (bFGF)	ReproCELL	Cat#RCHEOT002
Mitomycin C	Wako Pure Chemical Industries, Ltd.	Cat#139-18711
AccumaxTM	Nacalai Tesque	Cat#17087-54
StemPro-34 medium	Thermo Fisher Scientific	Cat#10640-019
Ascorbic acid	FUJIFILM Wako Pure Chemical Corporation	Cat#012-04802
1-thioglycerol	Sigma-Aldrich	Cat#M1753-100ML
Bone morphologic protein 4	R&D Systems	Cat#314-BP-010/CF
Activin A	R&D Systems	Cat#338-AC-010/CF
Vascular endothelial growth factor (VEGF)	FUJIFILM Wako Pure Chemical Corporation	Cat#229-01313
IWR-1	Sigma-Aldrich	Cat#I0161-5MG
IWP-2	Sigma-Aldrich	Cat#686770-61-6
Dulbecco’s modified Eagle’s medium (DMEM)	Nacalai Tesque	Cat#08458-45
Dulbecco’s Modified Eagle’s Medium (DMEM)-high glucose	Sigma-Aldrich	Cat#D5796
Iscove’s Modified Dulbecco’s Medium (IMDM)	Sigma-Aldrich	Cat#I3390
Fetal bovine serum (FBS)	Biosera	Cat#FB-1280/500
iMatrix-511Silk	MATRIXOME	Cat#387-10131
PDMS	Dow Corning	SYLGARD 184
D-PBS	TaKaRa	Cat#T900
Y-27632	Nacalai Tesque	Cat#034-24024
non-essential amino acid solution	Sigma-Aldrich	Cat#M7145
penicillin-streptomycin	Gibco	Cat#15140-122
L-glutamine	Gibco	Cat#ref. 25030-081
0.25% Trypsin-EDTA	Gibco	Cat#25200-072
Seahorse XF DMEM Medium, pH7.4	Agilent	Cat#103575-100
Seahorse XF 1.0M Glucose Solution	Agilent	Cat#103677-100
Triton X-100	Nacalai Tesque	Cat#9002-93-1
Tween 20	Nacalai Tesque	Cat#28353-14
iCell Cardiomyocytes Plating Medium	Fujifilm	Cat#M1001
iCell Cardiomyocytes Maintenance Medium	Fujifilm	Cat#M1003
Trizol Reagent	Invitrogen	Cat#108-95-2
E−4031	Sigma-Aldrich	Cat#M5060
Aspirin	Sigma-Aldrich	Cat#1044006
Ranolazine	Sigma-Aldrich	Cat#1598744
Mexiletin	Abcam	Cat#ab141823
Isoproterenol	Sigma-Aldrich	Cat#I5627
Metroprolol	Sigma-Aldrich	Cat#M5391
Verapamil	Sigma-Aldrich	Cat#V4629
Propranolol	Sigma-Aldrich	Cat#P0884
Dobutamine	Sigma-Aldrich	Cat#D0676
Milrinon	Wako	Cat#1504/10

**Experimental models: Cell lines**		

iCell^2^	Cellular Dynamics International (CDI)	Lot105451
Human Ventricular Cardiac Fibroblasts	Lonza	Cat#CC-2904
253G1 iPSC	Riken	HPS0002
Mouse embryonic fibroblast cells	ReproCELL	RCHEFC003

**Software and algorithms**		

FlowJo software	Treestar Inc.	N/A
Fiji: ImageJ	Fiji	https://fiji.sc/
MATLAB R2020	MathWorks	N/A
Origin2019	OriginLab	N/A
iDEP	Ge et al.^69^	http://bioinformatics.sdstate.edu/idep/
Metascape	Zhou et al.^70^	http://metascape.org/
EC50/IC50 value calculator	AAT Bioquest, Inc.	www.aatbio.com/tools/ic50-calculator

**Deposited data**		

TW and Control group data	This paper	GSE251914
Fetal and adult heart data	Kohjitani et al.^60^	GSE137255

**Critical commercial assays**		

Seahorse XF Cell Mito Stress Test Kit	Agilent Technologies	103015–100

**Others**		

Confocal microscope	Nikon	NIKON A1
Cell Motion Imaging System	SONY	SI8000
FACS Canto II flow cytometer	BD Biosciences	N/A
Transmission electron microscope	Hitachi Co.	H-7650
Extracellular flux analyzer	Agilent Technologies	Seahorse XFe 96
MEA data system	Multi Channel Systems	USB-ME64-System

### Resource availability

#### Lead contact

Further information and requests for resources and reagents should be directed to and will be fulfilled by the lead contact, Dr. Li Liu (liuli@ap.eng.osaka-u.ac.jp).

#### Materials availability

This study did not generate new materials.

#### Data and code availability

• All data reported in this paper will be shared by the lead contact upon request.
• The sequencing data analyzed in this study are deposited in GEO and are publicly available. Accession numbers are listed in the key resources table.
• This paper does not report original code.
• Any additional information required to reanalyze the data reported in this paper is available from the lead contact upon request.

### Experimental model and subject details

#### Cell lines

human iPSCs (253G1) were obtained from Riken, Japan and maintained in primate embryonic stem cell medium (ReproCELL, Kanagawa, Japan) with 5 ng/mL human basic fibroblast growth factor (bFGF; ReproCELL) on mouse embryonic fibroblast cells (ReproCELL) treated with mitomycin C (ReproCELL). Cryopreserved hiPSC-CMs (iCell,^2^ Lot:105451) were purchased from Cellular Dynamics International (CDI) (Madison, WI, USA). All the cell lines were free from mycoplasma contamination.

### Method details

#### Differentiation and culture of hiPSC-derived cardiomyocytes

Cardiomyocytes were differentiated from human iPSCs (253G1; Riken, Ibaraki, Japan), as described previously.^71^^,^^72^^,^^73^^,^^74^^,^^75^ Prior to differentiation, the hiPSCs were dissociated using an AccumaxTM (Nacalai Tesque, Kyoto, Japan) and transferred into a bioreactor (ABLE Corporation, Japan). Cardiac differentiation was initiated in StemPro-34 medium (Thermo Fisher Scientific, Waltham, MA, USA) containing 2 mM L-glutamine (Gibco), 50 μg/mL ascorbic acid (FUJIFILM Wako Pure Chemical Corporation, Osaka, Japan), and 400 μM 1-thioglycerol (Sigma-Aldrich, St. Louis, MO, USA). Several human recombinant proteins were also supplemented, including bone morphologic protein 4, activin A, bFGF (ReproCELL), vascular endothelial growth factor (VEGF; FUJIFILM Wako Pure Chemical Corporation), and small molecules (IWR-1 and IWP-2; Sigma-Aldrich). hiPSC-CMs were maintained in Dulbecco’s modified Eagle’s medium (DMEM; Nacalai Tesque, Kyoto, Japan) containing 10% fetal bovine serum (FBS; Biosera). In addition to the above mentioned 253G1, GCaMP3-positive human induced pluripotent stem cells (hiPSCs, 253G1) were generated, maintained and differentiated according to the previously published methods,^31^ the GCaMP3-253G1 CMs were used only for obtaining video showing the TW propagating within the tissue (Video S1). For the rest experiments, 253G1 CMs were used to avoid the effect of genetic modification on the CMs.

#### Device fabrication

The devices were prepared using a protocol modified from a previous report.^31^ A PDMS well (SYLGARD 184; Dow Corning, Midland, MI, USA) with an inner diameter of 8 mm was prepared using a tissue puncher. For the non-drug tests that do not require the microelectrode array (MEA), the well and a 3 mm PDMS pillar were aligned and attached to the bottom of 24-well plates (CellBIND, Corning, NY, USA) or MEAs (Multi Channel Systems, Reutlingen, Germany). For the drug assessment experiment, to use the costly MEA chips more efficiently, the iCTs were cultured in 24-well plates (Figure 1A) and transferred onto MEA chips only during signal recording. After recording, the MEA chips were washed and immediately reused for a new iCT. To achieve this, a layer of permeable PLGA fiber was prepared as previously reported^23^ and bonded to the bottom of the PDMS well and the 3 mm PDMS pillar. This permeable fiber scaffold provides support to the cardiac tissue during culture and allows recording by the MEA electrode during culture or drug assessments. After UV light treatment for 30 min, the PDMS wells were ready to be reused for cardiomyocyte culture.

#### Traveling wave tissue generation

The device was precoated with iMatrix-511Silk (MATRIXOME, Osaka, Japan) at 37°C for 30 min before cell plating. Single 253G1 cardiomyocytes were filtered using a 40-μm cell strainer (BD Falcon; Becton Dickinson, Franklin Lakes, NJ, USA) and resuspended at a density of 1 × 10^6^ cells/mL in culture medium containing 1% iMatrix-511Silk fragments, 3μM Y-27632 (Nacalai Tesque), 40% high glucose DMEM (Sigma-Aldrich), 40% IMDM (Sigma-Aldrich), 20% FBS (Gibco, USA), 1% minimum essential medium non-essential amino acid solution (Sigma-Aldrich), 0.1% penicillin-streptomycin (Gibco), and 0.5% L-glutamine (Gibco). Next, 2 × 10^5^ cells were plated in each PDMS well.

The iCell^2^ were thawed in a prepared medium (Plating Media, CDI). Since the purity of CMs in iCells^2^ is near 100%, to ensure the culture purity is similar to that of the 253G1-derived CM and to improve tissue adhesion, 20% Human Ventricular Cardiac Fibroblasts (NHCF-V, CC-2904, Lonza, Switzerland) was thawed and added to the iCell suspension. The iCells/NHCF-V mixture was resuspended in plating medium and 2 × 10^5^ cells were plated in each PDMS well.

After plating, the cardiomyocytes settled in the wells and formed a monolayer of tissue. The medium was changed to one containing 5% FBS on day 2. The sample size was determined according to previous reports which also included the *in vitro* experiments.^31^^,^^76^^,^^77^ On day 1 or 2, most of the samples contained TW. In order to define the control group, tissues with TW were randomly selected and the medium was exchanged with a 4°C medium to stop the TW. The devices were mounted on a rotary shaker (NA-M301; Nissin, Japan) to facilitate the exchange of oxygen and nutrients. The medium was then changed every 4 days. The fresh medium was preheated to 37°C before changing the medium. Similarly, the whole device was placed on a metal block preheated at 37°C during the medium change. The medium change was done gently to avoid disturbances to the traveling wave. All data in this study were collected after the tissues were cultured for 14 days. The TW was stopped by replacing the medium with 4°C medium; this will cause the CMs to stop beating for a while, and spontaneous beats would resume within one or two days.

#### Electrophysiological characterization

The field potentials of the CMs were recorded at 37°C using the MEA data system (USB-ME64-System, Multi Channel Systems, Germany). The data were collected and processed using MC_Rack (Multi Channel Systems). To obtain the activation map, the local activation time from a single electrode was obtained by calculating the minimum of the first-derivative plot of the raw curve. Linear interpolation between the electrodes was applied to calculate the isochronal map^23^^,^^78^ using the MATLAB function (MATLAB, MathWorks, USA). The field potential included the presence of a peak corresponding to the Na^+^ influx and depolarization, followed by the T wave determined as the repolarization phase corresponding to K^+^ efflux.^79^ The QT interval was obtained and corrected using the Fridericia’s correction formula: cQT interval = QT interval/ $\sqrt[{3}]{\text{RR}\;\text{interval}}$. To assess the effects of different drugs, 1 mL of fresh medium was added to the wells and maintained for 5 min before the baseline was recorded for 1 min. Then, another 1 mL of medium was added to twice the test concentration of the drug and gently pipetted into the well. Data were collected after 5 min. This process was repeated for all test concentrations. EC_50_/IC_50_ values were calculated using a web calculator (www.aatbio.com/tools/ic50-calculator).

#### Immunostaining and imaging

Tissues were fixed in 4% paraformaldehyde (PFA) for 0.5 h, permeabilized with 0.5% (v/v) Triton X-100 in Dulbecco’s PBS (D-PBS) for 1 h, and immersed in blocking solution at 4°C overnight. The tissues were then incubated with the primary antibodies anti-α-actinin (1:1000; A7811; Sigma-Aldrich), anti-troponin T2 (TnT2; 1:200; SC-20025; Santa Cruz Biotechnology, Dallas, TX, USA), anti-connexin 43 (Cx43; 1:200; C6219; Sigma-Aldrich), or anti-β-MHC (1:100; SC-53089; Santa Cruz Biotechnology) at 4°C overnight. Thereafter, the tissues were rinsed with PBS and incubated with the secondary antibodies Alexa Fluor 594 anti-mouse IgG (715-586-150; Jackson Immuno Research, West Grove, PA, USA), DyLight-594 anti-mouse IgM (715-516-020; Jackson Immuno Research), Alexa Fluor 647 anti-rabbit IgG (A21245; ThermoFisher), and Alexa Fluor 488 anti-rabbit IgG (A21206; ThermoFisher) at a dilution of 1:300 in blocking buffer at room temperature for 1 h. DAPI (300 nM; Wako Pure Chemical Industries, Ltd.) was used to stain the nuclei for 30 min. Images were captured using a confocal microscope (NIKON A1; Nikon).

#### Transmission electron microscopy (TEM)

The iCTs were observed using a transmission electron microscope (H-7650; Hitachi Co., Tokyo, Japan). Specimens for TEM were prepared according to the following procedure: tissues were fixed with 2.5% glutaraldehyde for 120 min. The samples were then post-fixed with 1% osmium tetraoxide for 90 min and dehydrated through a graded series of ethanol (50%–100%) and propylene oxide. The tissues were then embedded in epoxy resin, sliced using an ultramicrotome (Ultracut E; Reichert-Jung, Vienna, Austria), and stained with uranyl acetate and lead citrate.

#### Mitochondrial respiration assay

Mitochondrial function was analyzed using a Seahorse XF96 extracellular flux analyzer (Agilent Technologies, Carlsbad, CA, USA). After culturing for 14 days, the hiPSC-CM tissue was dissociated into a single-cell suspension using 0.25% trypsin/EDTA (Thermo Fisher) and then seeded in a microplate (XF96, Agilent Technologies) at a density of 2×10^4^ cells/well. After 3 days of culture, the culture medium was replaced with base medium (Seahorse XF assay media; Agilent Technologies, Carlsbad, CA, USA) supplemented with 1 mM sodium pyruvate. Substrates and inhibitors were added during measurements to attain a final concentration of 3.5 μM 4-(trifluoromethoxy) phenylhydrazone (FCCP; Seahorse Bioscience, Billerica, MA, USA), 1 μM oligomycin, 0.5 μM antimycin, and 0.5 μM rotenone for the MitoStress assay.

#### Flow cytometry

After differentiation, hiPSC-CMs were dissociated into single cells, fixed in 4% PFA for 0.5 h, and permeabilized with Triton X-100 (0.5% v/v in D-PBS) for 0.5 h at room temperature. The cells were then incubated with anti-TnT2 antibodies (SC-20025; 1:200; Santa Cruz Biotechnology) or isotype-matched antibodies (557782; BD Phosphoflow) at 37°C for 0.5 h, washed with D-PBS, and incubated with Alexa Fluor 488 anti-mouse IgG (715-546-150, 1:500; Jackson Immuno Research). The cells were then washed twice with D-PBS. Flow cytometry was performed using a FACS Canto II flow cytometer (BD Biosciences, USA) and analyzed using FlowJo software (Treestar Inc., USA).

#### Western blot

The iCT was washed with PBS and lysed with lysis buffer [1% CHAPS, 25 mmol/L Tris-HCl (pH 7.4), 137 mmol/L NaCl, 2.68 mmol/L KCl, and 5 mmol/L EDTA]. Protein concentration was determined using a BCA Protein Assay Kit (Thermo). Lysate samples were mixed with 4 × loading sample buffer (Bio-Rad) and mercaptoethanol (2.5%). Proteins were separated by SDS-PAGE and transferred onto polyvinylidene fluoride (PVDF) membranes. After blocking in 3% non-fat milk for 1 h, the transferred membrane was incubated with primary antibodies at 4°C overnight and thereafter with secondary antibodies at room temperature for 0.5 h. The membrane signals were recorded using ECL prime reagent (GE). Protein expression data were quantified using an ImageQuant LAS 4000 (GE) system.

#### Cell motion analysis

Contractile properties were evaluated using a Cell Motion Imaging System (SI8000; SONY, Tokyo, Japan). iCTs, with or without TW, were cultured for 14 days. The TWs were stopped in the TW group at least 2 days before recording to allow the restart of spontaneous beats. Videos were recorded at a frame rate of 150 frames per second, a resolution of 1024 × 1024 pixels, and a depth of 8 bits.

#### RNA sequencing

Library preparation was performed using the TruSeq stranded mRNA sample prep kit (Illumina, San Diego, CA, USA) according to the manufacturer’s instructions. Sequencing was performed on an Illumina NovaSeq 6000 platform using the 100 bp paired-end mode. Sequenced reads were mapped to the human reference genome sequences (hg19) using TopHat v2.0.13 in combination with Bowtie2 ver. 2.2.3 and SAMtools ver. 0.1.19. Fragments per kilobase of exon per million mapped fragments (FPKMs) were calculated using Cufflinks ver. 2.2.1. The PCA analysis and hierarchical clustering were performed by using iDEP (http://bioinformatics.sdstate.edu/idep/). The GO enrichment analysis was performed by using Metascape (http://metascape.org/). The Control group and TW group data in this paper could be visited under the accession number GEO: GSE251914. The fetal and adult heart data were downloaded from the NCBI GEO databank (GEO: GSE137255).^60^ To compare the gene expression in the present work with fetal and adult heart data in a previous report,^60^ the FPKM data were converted into Transcripts Per Million (TPM).

### Quantification and statistical analysis

All quantitative data are presented as the mean ± standard error of the mean (Mean ± SEM). The differences among different groups were analyzed using unpaired Student’s *t* test (between two groups) or ANOVA (one-way analysis of variance), followed by Tukey’s post hoc test (among three or more groups). p < 0.05 was considered statistically significant.
