# Supplementary material for: Human induced pluripotent stem cell-derived closed-loop cardiac tissue for drug assessment
Source: iScience. 2024 Jan 23;27(2):108992. doi: 10.1016/j.isci.2024.108992 (PMC10850789; doi:10.1016/j.isci.2024.108992)
Supplement: Document S1. Figures S1–S8 [file mmc1.pdf]

iScience, Volume *27*

Supplemental information

Human induced pluripotent stem cell-derived closed-loop cardiac tissue for drug assessment

Junjun Li, Ying Hua, Yuting Liu, Xiang Qu, Jingbo Zhang, Masako Ishida, Noriko Yoshida, Akiko Tabata, Hayato Miyoshi, Mikio Shiba, Shuichiro Higo, Nagako Sougawa, Maki Takeda, Takuji Kawamura, Ryohei Matsuura, Daisuke Okuzaki, Toshihiko Toyofuku, Yoshiki Sawa, Li Liu, and Shigeru Miyagawa

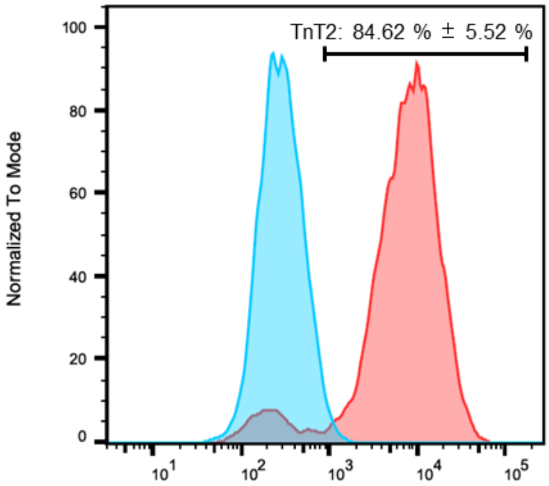

Figure S1. Culture of hiPSC-derived close-loop cardiac tissue (iCT), related to Figure 1. Flow cytometry data of cTnT positive cell (hiPSC cell line: 253G1) on day 0, Mean ± SEM, n = 3 independent experiments.

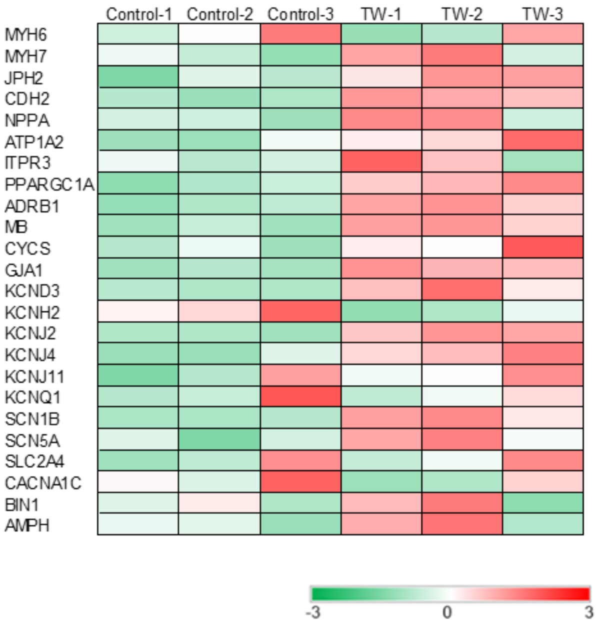

Figure S2. Heatmaps showing the expression of cardiac maturation-specific genes, related to Figure 2. FPKM data were used to generate the heatmap.

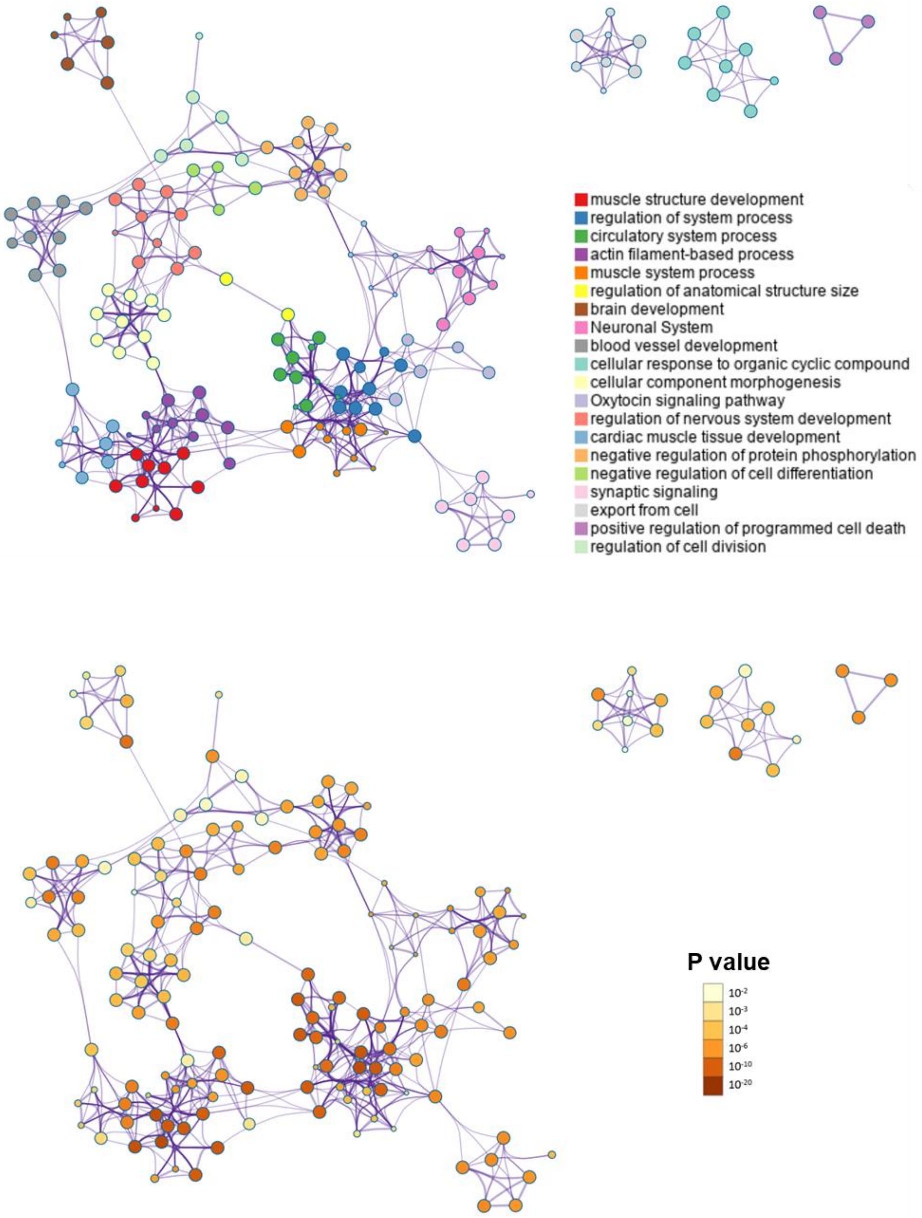

Figure S3. Network of enriched terms, related to Figure 2. (top) Coloured by cluster ID, where nodes that share the same cluster ID are typically close to each other. (bottom) Coloured by *p*-value, where terms containing more genes tend to have a more significant p-value

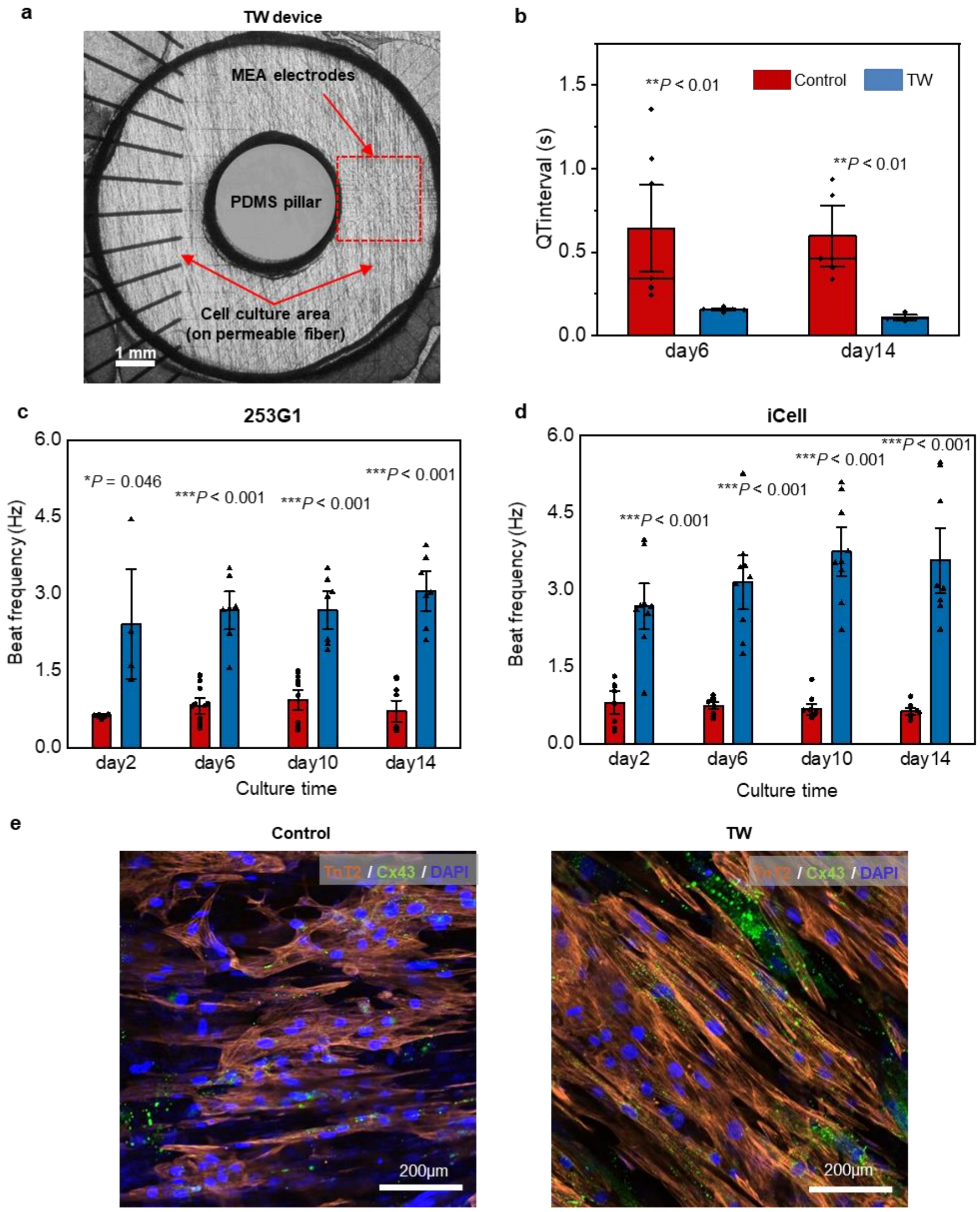

Figure S4. hiPSC-derived closed-loop cardiac tissue (iCT) on permeable fibers for MEA recording, related to Figure 6. (a) Image of iCT device on MEA. The permeable fiber will allow the culture of cells, while allowing the recording of electrical signals by MEA. (b) QTinterval of 253G1-made iCT at different culture times (Mean ± SEM; Day6: Control: n = 7; TW: n = 8; Day14: Control: n = 5; TW: n = 4 independent biologically

samples from four differentiations) (c,d) Beat rates of 253G1-made iCT (b, c) and iCell-made iCT (d) on permeable fiber at different culture times (Mean ± SEM; 253G1-derived cardiomycytes: Control: n = 14; TW: n = 7; iCell: Control: n = 8; TW: n = 9 independent biologically samples from 253G1: three differentiations; iCell: Lot: 105451). *P < 0.05, **P < 0.01, ***P < 0.001 (Student's t-test). (e) iCT (253G1) on permeable fiber with or without TW on day 14. Cardiomyocytes were stained with anti-TnT2, anti-Cx43, and DAPI.

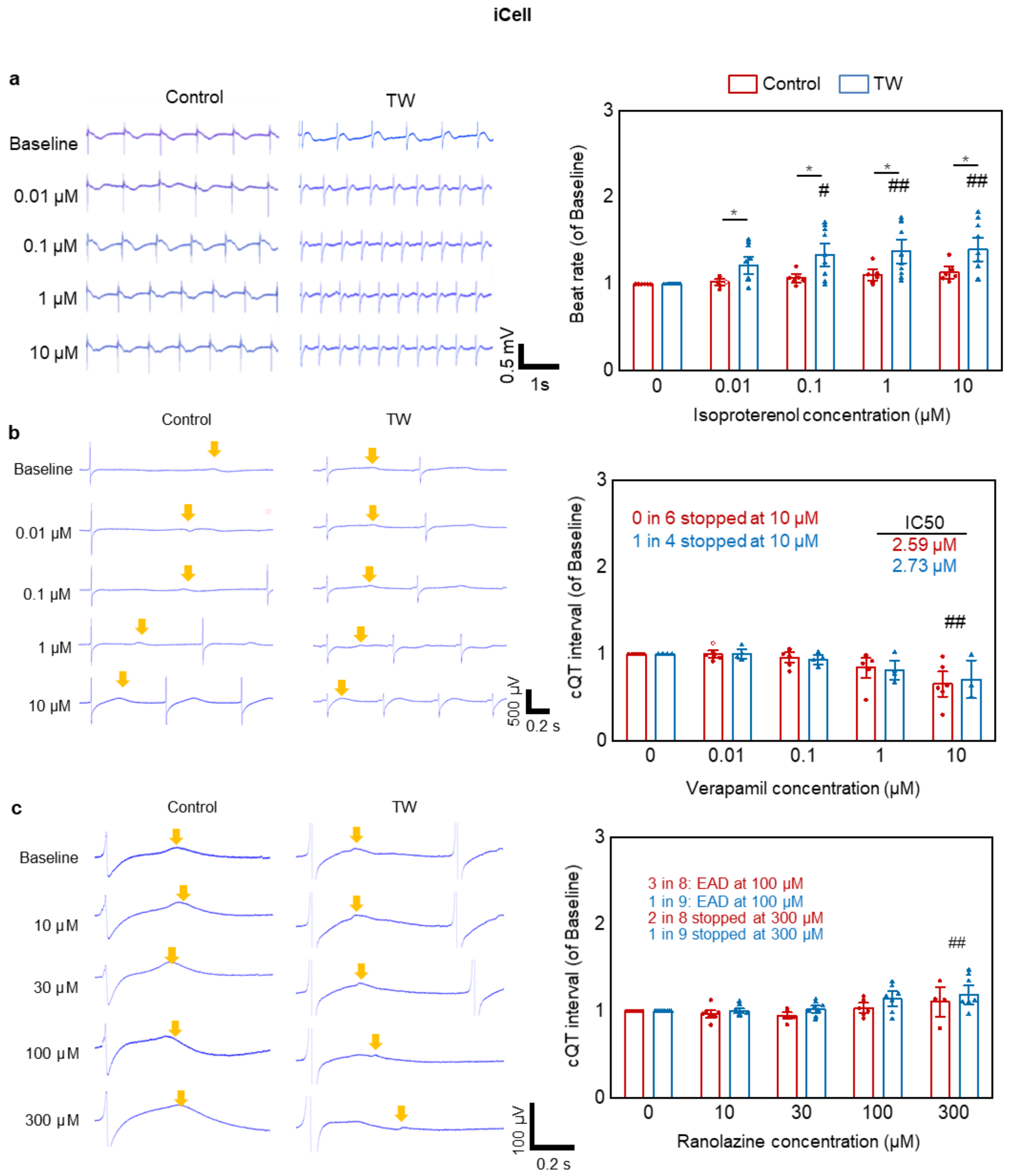

Figure S5. Drug response of hiPSC-derived closed-loop cardiac tissue (iCT) with or without traveling wave (TW) training, related to Figure 6. (a-c) Representative trace (left) and drug effect (right) of CMs (iCell) treated with isoproterenol (β adrenoceptor agonist), verapamil (calcium blocker, low TdP Risk), and ranolazine (sodium and hERG blocker, low TdP risk) (Mean ± SEM; isoproterenol: Control: n = 6; TW: n = 10; verapamil: Control: n = 6; TW: n = 4; ranolazine: Control: n = 8; TW: n = 9; biologically

independent samples from iCell Lot: 105451). * $P < 0.05$ (Student's *t*-test); # $P < 0.05$, ## $P < 0.01$ vs. values before drug treatment (ANOVA). The yellow arrows mark the Twave.

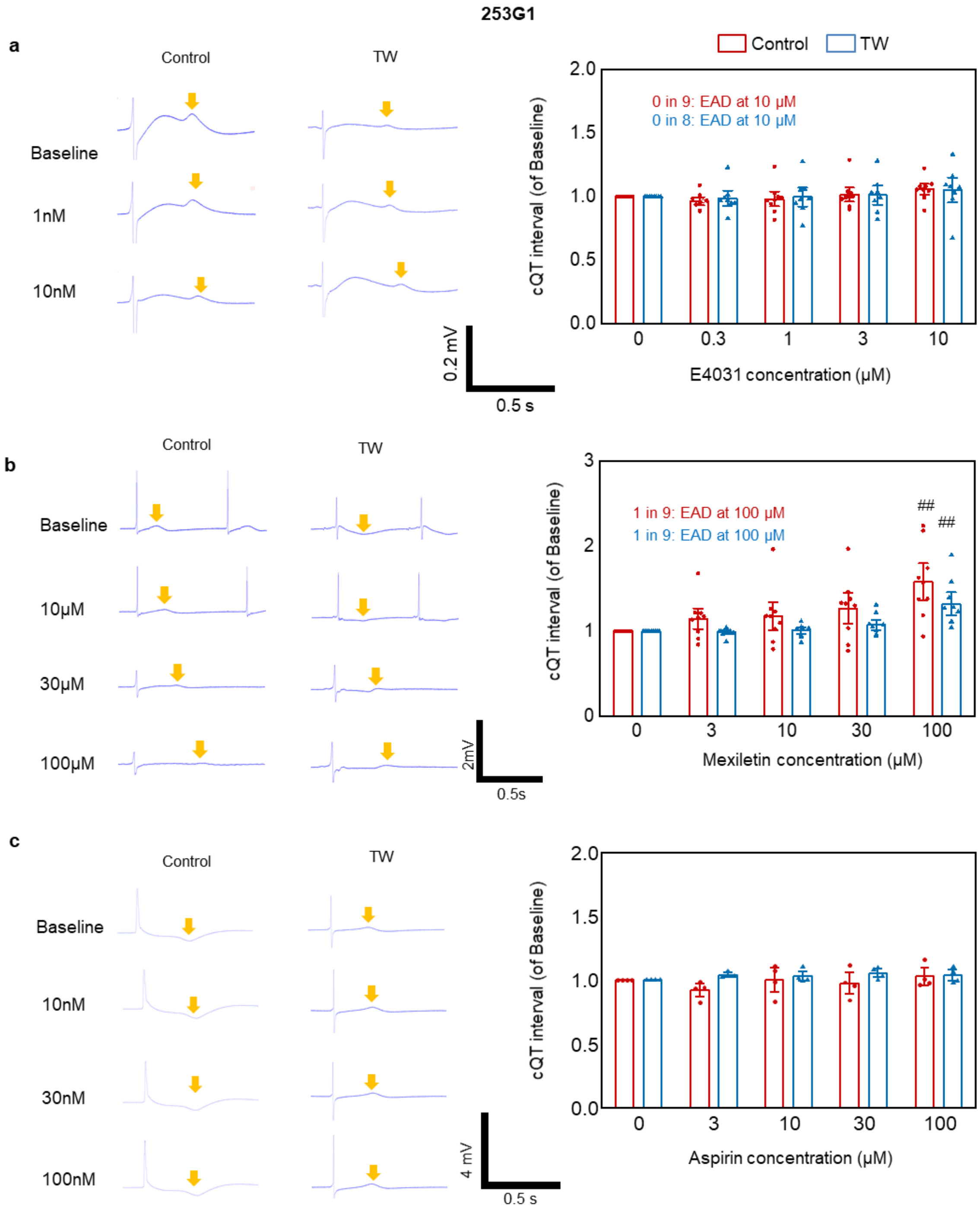

Figure S6. Drug response of hiPSC-derived closed-loop cardiac tissue (iCT) with or without traveling wave (TW) training, related to Figure 6. Representative trace (left) and drug effect (right) of CMs (253G1) treated with E4031 (potassium blocker), mexiletine (sodium channel and hERG channel blocker), and aspirin (negative control) (Mean ± SEM; E4031: Control: n = 9; TW: n = 8; mexiletine: Control: n = 9; TW: n = 9; aspirin: Control: n = 4; TW: n = 4; biologically i7ndependent samples from two to four differentiations). ## $P < 0.01$ vs. values before drug (ANOVA). The yellow arrows mark the Twave.

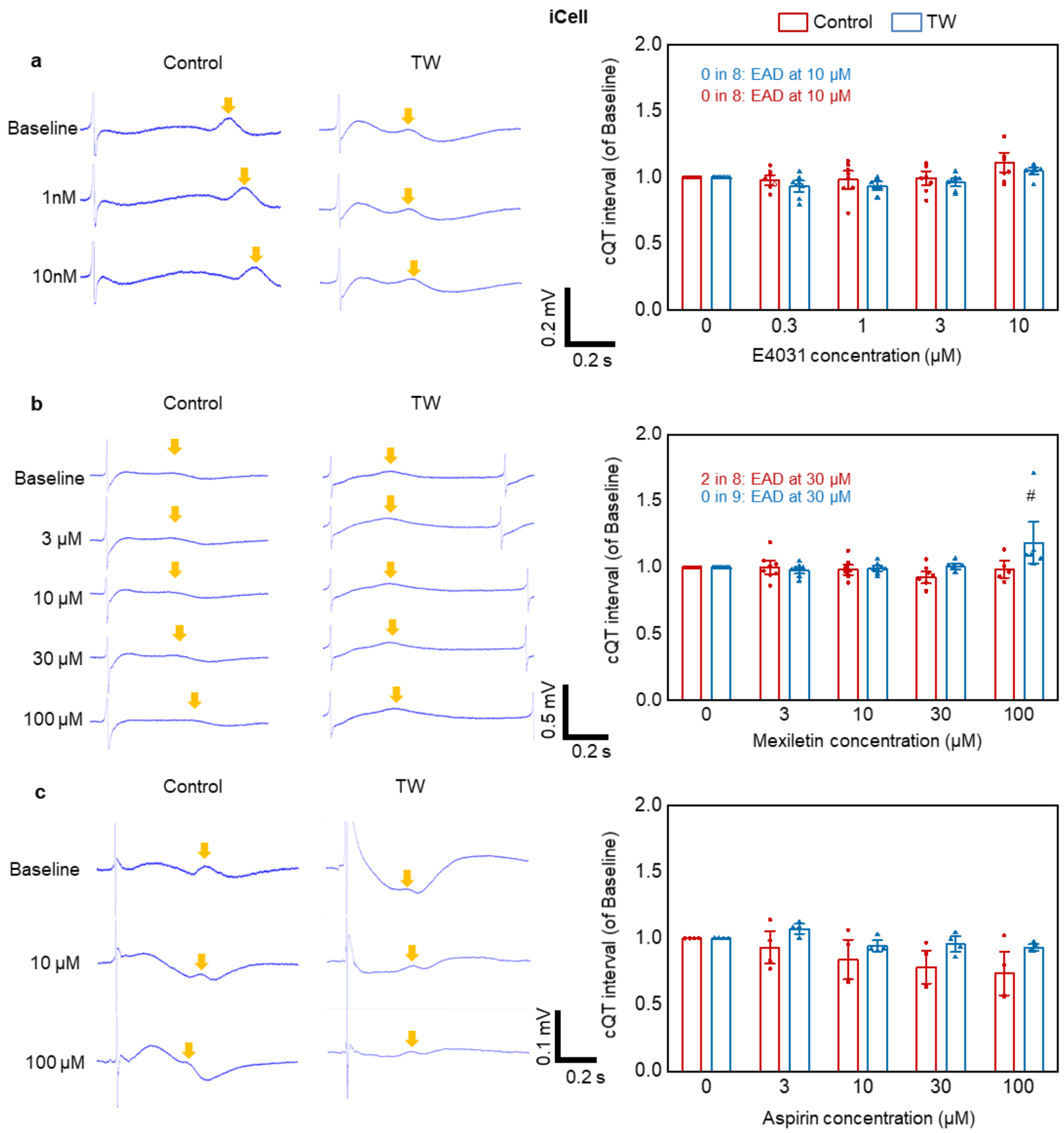

Figure S7. Drug response of hiPSC-derived closed-loop cardiac tissue (iCT) with or without traveling wave (TW) training, related to Figure 6. Representative trace (left) and drug effect (right) of CMs (iCell) treated with E4031 (potassium blocker), mexiletine (sodium channel and hERG channel blocker), and aspirin (negative control) (Mean ± SEM; E4031: Control: n = 8; TW: n = 8; mexiletine: Control: n = 8; TW: n = 9; aspirin:

Control: n = 4; TW: n = 4; biologically independent samples from iCell Lot: 105451). ## $P < 0.01$ vs. values before drug treatment (ANOVA). The yellow arrows mark the Twave.

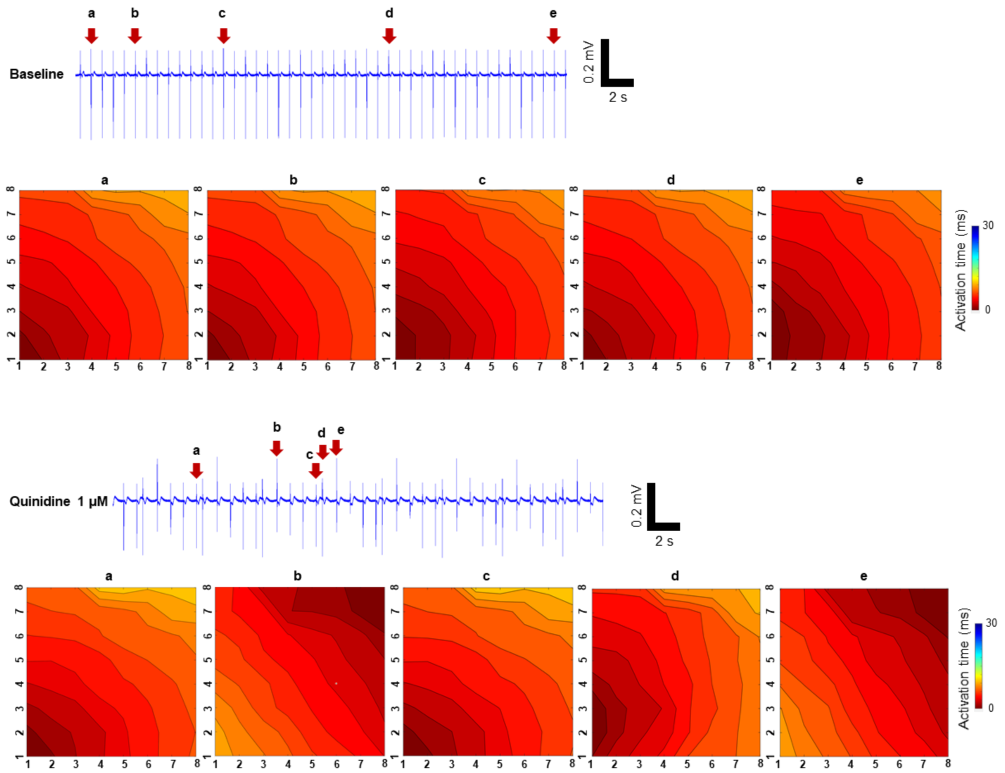

Figure S8. Representative trace and activation map of CMs (253G1) treated without and with quinidine (high TdP risk), **related to Figure 7**. The red arrows indicated the moments when the activation maps were recorded.
